# Administration of Secretome Derived from Human Mesenchymal Stem Cells Induces Hepatoprotective Effects in Models of Idiosyncratic Drug-Induced Liver Injury Caused by Amiodarone or Tamoxifen

**DOI:** 10.3390/cells12040636

**Published:** 2023-02-16

**Authors:** Ya-Lin Huang, Cristian De Gregorio, Verónica Silva, Álvaro A. Elorza, Patricio Léniz, Víctor Aliaga-Tobar, Vinicius Maracaja-Coutinho, Mauricio Budini, Fernando Ezquer, Marcelo Ezquer

**Affiliations:** 1 Centro de Medicina Regenerativa, Instituto de Ciencias e Innovación en Medicina, Facultad de Medicina, Clínica Alemana-Universidad del Desarrollo, Santiago 7610658, Chile; 2 Instituto de Ciencias Biomédicas, Facultad de Medicina y Ciencias de la Vida, Universidad Andres Bello, Santiago 7610658, Chile; 3 Unidad de Cirugía Plástica, Reparadora y Estética, Clínica Alemana, Santiago 7610658, Chile; 4 Advanced Center for Chronic Diseases (ACCDiS), Facultad de Ciencias Químicas y Farmacéuticas, Universidad de Chile, Santiago 7610658, Chile; 5 Centro de Modelamiento Molecular, Biofísica y Bioinformática (CM2B2), Facultad de Ciencias Químicas y Farmacéuticas, Universidad de Chile, Santiago 7610658, Chile; 6 Laboratorio de Bioingeniería, Instituto de Ciencias de la Ingeniería, Universidad de O’Higgins, Rancagua 7610658, Chile; 7 Instituto de Investigación en Ciencias Odontológicas, Facultad de Odontología, Universidad de Chile, Santiago 7610658, Chile

**Keywords:** drug-induced liver injury, hepatic regeneration, amiodarone, tamoxifen, cell free therapy

## Abstract

Drug-induced liver injury (DILI) is one of the leading causes of acute liver injury. While many factors may contribute to the susceptibility to DILI, obese patients with hepatic steatosis are particularly prone to suffer DILI. The secretome derived from mesenchymal stem cell has been shown to have hepatoprotective effects in diverse in vitro and in vivo models. In this study, we evaluate whether MSC secretome could improve DILI mediated by amiodarone (AMI) or tamoxifen (TMX). Hepatic HepG2 and HepaRG cells were incubated with AMI or TMX, alone or with the secretome of MSCs obtained from human adipose tissue. These studies demonstrate that coincubation of AMI or TMX with MSC secretome increases cell viability, prevents the activation of apoptosis pathways, and stimulates the expression of priming phase genes, leading to higher proliferation rates. As proof of concept, in a C57BL/6 mouse model of hepatic steatosis and chronic exposure to AMI, the MSC secretome was administered endovenously. In this study, liver injury was significantly attenuated, with a decrease in cell infiltration and stimulation of the regenerative response. The present results indicate that MSC secretome administration has the potential to be an adjunctive cell-free therapy to prevent liver failure derived from DILI caused by TMX or AMI.

## 1. Introduction

Liver regeneration is essential for tissue survival upon acute and chronic exposure to toxins and drugs [1]. Nevertheless, some pathological conditions can overwhelm and inhibit its intrinsic regenerative potential, facilitating the progression of injury into a state of functional impairment, leading to consequences that include liver failure and death [2].

Drug-induced liver injury (DILI) is one of the leading causes of acute liver injury, accounting for 13% of cases of acute liver failure (ALF) [3,4]. Many factors may contribute to the susceptibility of a patient to DILI, such as underlying chronic liver disease, and alcohol intake [5]. Several studies have demonstrated that patients with nonalcoholic fatty liver disease (NAFLD) have impaired liver regeneration [6,7]. Therefore, obese patients are more prone to suffer DILI due the intrinsic susceptibility of their damaged liver [8,9,10,11].

The lack of therapeutic alternatives for DILI requires the development of new therapeutic agents. Multipotent mesenchymal stromal cells (MSCs), are a population of self-renewable and undifferentiated cells, present in multiple mesenchymal tissues, including adipose tissue, with great potential for the regeneration of injured tissues [12]. In this regard, multiple studies, have described the potential role of MSCs in promoting liver regeneration, even in special pathological conditions with impaired hepatic regeneration, such as NAFLD [13,14,15,16].

It has been shown that the therapeutic effect of MSC administration can be attributed to transient paracrine effects, which include the release of soluble factors and extracellular vesicles, which constitute the MSC secretome [12,14,17,18,19].

Amiodarone (AMI) is a potent frequently prescribed class III antiarrhythmic drug, which has been in use since the 1960s [20]. Despite its beneficial properties, AMI has several adverse effects including hepatic steatosis, steatohepatitis, and even cirrhosis [21]. The mechanisms through which AMI induces liver damage are not completely clear; however, hepatitis induced by AMI has been proposed to be the result of mitochondrial impairment due to the uncoupling of oxidative phosphorylation from electron transport, with inhibition of fatty acid β-oxidation [22,23].

Tamoxifen (TMX) is the most commonly used drug for the treatment of estrogen receptor positive breast cancer [24]. However, steatosis and steatohepatitis are frequent side effects in breast cancer patients undergoing TMX treatment. Moreover, some patients also develop fibrosis and hepatic necrosis [25,26]. Although the exact mechanisms involved in liver toxicity induced by TMX remain controversial, a direct impact on lipid metabolism, β-oxidation, and mitochondrial membrane potential has been reported [27,28,29].

The predominance of hepatocytes in terms of abundance and functional contribution to the liver has led to propose primary human hepatocytes (PHHs) cultures as the gold standard to test toxicity and to develop therapeutic tools. However, the use of PHH is greatly hindered because human liver samples are scarce, PHHs show in vitro instability, and high batch-to-batch variations in their content of cytochrome (CY) P450 enzymes, which are directly related to cytotoxicity [30,31].

To circumvent these drawbacks, several hepatic cell models have been developed for drug metabolism, hepatotoxicity studies, and therapeutic screening [32].

HepG2 cells have been used extensively as a valuable preclinical model for the evaluation of drug toxicity [33,34]. Compared with PHH, HepG2 cells have a low expression of CYP450 enzymes [35,36]. Nevertheless, some studies have shown that HepG2 cells exhibit similar CYP 1A2 and 3A4 inducibility in relation to human hepatocytes, together with a complete set of phase II enzymes [37,38,39]. Moreover, in terms of detecting mitochondrial toxicity, diverse studies present HepG2 cells as advantageous over primary human hepatocytes [40,41].

On the other hand, the HepaRG cell line is considered a surrogate of primary human hepatocytes for DILI applications and therapeutics testing, since it reproduces hepatocyte-like characteristics, including more abundant CYP450 enzymes compared to HepG2 cells [42,43,44]. However, their utility for toxicity screening that involves large sets of compounds and their entire metabolism has not been extensively examined. For this reason, we think that the evaluation of the hepatoprotective effect of MSC secretome on both cell lines would be complementary.

The in vitro study of the therapeutic effects of MSC secretome on DILI can be controlled more closely than in vivo approaches. However, the selected conditions may not well enough reflect those present in vivo [45]. Although only a few drug-metabolizing enzymes have been reported in other hepatic cell types, such as endothelial cells, Kupffer cells, or hepatic stellate cells (HSCs), their direct or indirect role in DILI has been well documented. For example, reactive oxygen species and lipid peroxidation products activate Kupffer and HSCs, playing a role in the inflammatory response and liver fibrosis, respectively [46,47]. In that sense, both DILI and some hepatic conditions related to DILI, such as hepatic steatosis, can be experimentally induced in rodents via the chronic exposure to drugs or a high-fat diet (HFD), respectively [48,49]; the synergic effect of both conditions has already been shown [50,51].

The goal of the present work was to evaluate the hepatoprotective potential of the secretome of MSCs derived from adipose tissue to prevent DILI induced by AMI or TMX. First, the effect of the MSC secretome was studied in two complementary cell models, HepG2 and HepaRG cells, and then the results were validated in an in vivo mouse model of DILI.

## 2. Materials and Methods

All compounds and reagents used in this work were of analytical grade. Stock solutions of amiodarone hydrochloride (AMI) (Sigma-Aldrich A8423, Saint Luis, MI, USA) and tamoxifen (TMX) (Sigma T5648, Sigma-Aldrich) were prepared in dimethyl sulfoxide (DMSO) and the final solvent concentration was below 0.5%. As a control group, in each experiment, cells were incubated with the drug solvent (0.5% DMSO).

### 2.1. Isolation, Expansion and Characterization of MSCs Derived from Human Adipose Tissue

MSCs were isolated from subcutaneous adipose tissue (abdominal region) obtained from donors undergoing cosmetic liposuction at CAS-UDD, Chile, as previously described [52]. Written informed consent was obtained for all samples. All protocols were approved by the Ethics Committee of Facultad de Medicina, CAS-UDD. After, two subcultures cells were characterized as was previously described [52].

### 2.2. MSC Secretome Preparation

MSC secretome was obtained as previously described [53]. MSCs (passage 3) were allowed to grow up to 80% confluence in alpha minimal essential medium (α-MEM) (Gibco, Carlsbad, CA, USA), plus 10% fetal bovine serum (FBS) (Hyclone, Marlborough, Australia) and 0.16 mg/mL gentamicin (Sanderson, Laurel, MS, USA). Then, cells were washed thoroughly with phosphate-buffered saline (PBS) and incubated for 48 h with serum-free α-MEM without phenol red. The culture medium (secretome) was collected and concentrated (30 times *v*/*v*) by 3kDa cutoff filters (Millipore, Burlington, MA, USA). The protein concentration was determined, and the secretome was frozen at −80 °C until use.

### 2.3. Cell Lines and Cell Culture

HepG2 cells were purchased from the European Collection of Cultures (ECCA N°85011430) and were maintained as an adherent cell line in Dulbecco’s modified Eagle’s medium (DMEM) (Gibco, Carlsbad, CA, USA) supplemented with 10% FBS, 2 mmol/L GlutaMAX-1 (Thermo Fisher, Waltham, MA, USA) and 0.16 mg/mL gentamicin.

HepaRG cells were previously differentiated by the manufacturer. Media for the maintenance and chemical treatment of HepaRG cells were prepared in accordance with the manufacturer’s instructions in William’s Medium E. HepaRG cells were seeded using HepaRG Thaw, Plate, & General Purpose Medium supplemented with 2 mmol/L GlutaMAX-1 and 0.16 mg/mL gentamicin to restore hepatocyte function. Twenty-four hours prior to testing, the medium was replaced with HepaRG Tox Medium Supplement with 2 mmol/L GlutaMAX-1 and 0.16 mg/mL gentamicin to increase the capacity of drug-metabolizing enzymes.

HepaRG Thaw, Plate, & General Purpose Medium Supplement; HepaRG Tox Medium Supplement; William’s Medium E, and GlutaMAX-1, were purchased from Thermo Fisher Scientific.

Both cell lines were incubated at 37 °C in a 5% CO_2_ atmosphere, and cells were passaged as needed using 0.25% trypsin-EDTA (Gibco). For drug toxicity testing, HepG2 or HepaRG cells were seeded at a density of 30,000 cells in collagen-coated 96-well plates and were allowed to adhere for 24 h prior to treatment with drugs or vehicle. The following day, cells were treated with test compounds dissolved in culture medium.

For all toxicity tests, three independent experiments were performed in triplicate for each experimental condition and its control.

### 2.4. RNA Isolation and Gene Expression Analysis

The mRNA level of the genes of interest was assessed by RT-qPCR as previously described [13]. After treatment, total RNA was purified from cells seeded in a 12-well plate using TRIzol (Invitrogen, Waltham, MA, USA) following the manufacturer’s instructions. Two micrograms of total RNA were used to perform reverse transcription with MMLV reverse transcriptase (Invitrogen) and oligo dT primers. CYP 1A1, 1A2, 2B6, 2C9, 2C19, 2D6, 2E1, 3A4, 7A1, TNF-α, IL-6, inducible nitric oxide (iNOS), and alpha-smooth muscle actin (α-SMA) cDNAs were amplified with specific primers (Table S1) using a Light-Cycler 1.5 thermocycler (Roche, Rotkreuz, Switzerland). Relative quantification was performed by the ΔΔCT method [54]. The mRNA level of each target gene was normalized against the mRNA level of ribosomal protein L13a (RPL13a) and GAPDH and expressed as fold of change versus control cells (0.5% DMSO).

### 2.5. Evaluation of Cell Viability

We used a validated method to screen for AMI and TMX toxicity based on staining cells with 3-[4,5-dimethylthiazol-2-yl]-2,5-diphenyl-tetrazolium bromide (MTT) [34,55]. This method assesses the ability of viable cells to form MTT formazan by the mitochondrial enzyme succinate dehydrogenase.

Drug cytotoxicity assay: HepG2 and HepaRG cells in the logarithmic growth phase were plated in a 96-well plate and incubated for 24 h in fresh medium. Then, they were treated with increasing concentrations of AMI (10 to 90 µM) or TMX (5 to 25 µM) for 24 h at 37 °C, while cells incubated in the presence of 0.5% DMSO served as the control group. MTT (0.5 mg/mL) was added to each well and the reaction was stopped 4 h later by adding 150 µL of DMSO. The optical density of each well was measured at 570 nm using a Tecan microculture plate reader.

Cell viability was expressed as the ratio of cells exposed to different drug concentrations to control cells treated with the drug solvent (0.5% DMSO).

Effect of MSC secretome on the viability of hepatic cells: to ascertain whether the MSC secretome has cytotoxic effects, HepG2 and HepaRG cells were seeded in medium supplemented with 0, 20, 40, and 100 µg/mL of MSC secretome. Cell viability was evaluated after 24 h using the MTT assay.

In vitro hepatoprotective effect of MSC secretome: HepG2 and HepaRG cells were exposed to AMI (20 and 50 µM for HepG2, 10 and 15 µM for HepaRG) or TMX (15 and 20 µM), alone or with two concentrations of MSC secretome (20 and 70 µg/mL) for 24 h at 37 °C. The same volume of PBS was used as vehicle (secr vehicle group) to compare with MSC secretome-treated cells. Cell viability was evaluated with the MTT assay.

### 2.6. Evaluation of Cytotoxicity (Lactate Dehydrogenase Release)

Cytotoxicity was determined using the CytoTox-ONE Assay (Promega, Madison, WI, USA). This assay measures the release of lactate dehydrogenase (LDH), a marker for loss of integrity of the cell membrane.

Briefly, HepG2 or HepaRG cells were seeded at a density of 30,000 cells/well in collagen coated 96-well plates and were allowed to adhere for 24 h prior to treatment. The following day, cells were treated with AMI or TMX, alone or with MSC secretome (20 µg/mL).

After treatment for 24 h, 100 µL of assay buffer were added to 100 µL of supernatant from the treated cell culture medium and incubated at 22 °C for 10 min. Fluorescence was recorded in a Turner BioSystems Modulus fluorometer (Turner BioSystem Inc., Sunnyvale, CA, USA) (ex/em 560 nm/590 nm). LDH release was expressed as the ratio of the fluorescence of treated cells to that of DMSO controls.

### 2.7. Determination of Caspase 3/7 Activity

Caspase-3 and caspase-7 are both activated during apoptosis and play an important role in the intrinsic and extrinsic pathways of apoptosis. Caspase 3/7 activity was assayed in HepG2 and HepaRG cells using the Apo-ONE Homogeneous Caspase-3/7 Assay (Promega), according to the manufacturer’s manual. Briefly, HepG2 or HepaRG cells were seeded at a density of 30,000 cells/well in collagen coated black 96-well plates, and were allowed to adhere for 24 h prior to treatment. The following day, cells were treated with AMI or TMX, alone or with MSC secretome (20 µg/mL), for 24 h and washed twice with PBS. A volume of 50 µL of PBS was added to each well plus 50 µL of homogeneous caspase 3/7 reagent. The plates were incubated for 1 h at room temperature (RT) and fluorescence was recorded in a Turner BioSystems Modulus fluorometer (ex/em 490 nm/520 nm).

### 2.8. Evaluation of Cytochrome C Release

The apoptotic stimulus triggers the release of cytochrome C from mitochondria into the cytosol where it activates caspase-3.

Detection of cytochrome C by fluorescence microscopy: HepG2 or HepaRG cells were seeded at a density of 350,000 cells per well in microscopic cover glasses placed in 24-well plates, and allowed to adhere for 24 h prior to treatment. The following day, cells were treated with AMI or TMX, alone or with MSC secretome (20 µg/mL), for 24 h. Cells were stained with 50 nM Mitotracker Red CMXRos (Invitrogen), and crosslinked with 4% formaldehyde in PBS. Permeabilization was performed with 0.2% Triton X-100 followed by blocking with 5% bovine serum albumin (BSA). Immunostaining was performed using an anti-cytochrome C antibody (mouse #MA511823, Thermo Fisher; secondary antibody: anti-mouse Alexa Fluor 488 #A11001, Thermo Fisher), while the nuclei were counter-stained with 4′-6′-diamino-2-phenylindole (DAPI). The colocalization analysis was performed by confocal microscopy (Fluoview FV101, Olympus, Shinjuku, Japan) [56].

Detection of cytochrome C by flow cytometry: the selective permeabilization of the plasma membrane allows cytoplasmic cytochrome C to diffuse out of the cell, leading to a low content of cytochrome C in the cell. A semiquantitative evaluation of cytochrome C release was performed as previously described [57]. HepG2 and HepaRG cells were treated as described above and 350,000 cells per condition were harvested and permeabilized with 10 µg/mL digitonin (Merck, Rahway, NJ, USA) in PBS without calcium at RT for 20 min. Cells were fixed in 4% paraformaldehyde for 20 min at RT and washed with blocking buffer (BSA 10% in PBS) for 1 h and incubated overnight at 4 °C with monoclonal anti-cytochrome C antibody (CTC03-2B5, Thermo Fisher) in blocking buffer. Cells were washed with blocking buffer and incubated for 1 h with a secondary antibody (A31571, Invitrogen) labeled with Alexa Fluor 488. After an additional washing step with PBS, the fluorescence signal of the cell suspensions was acquired in a Cyan ADP flow cytometer. Data were analyzed with the Summit v4.3 software, (Cyan, Mead, WA, USA).

### 2.9. Determination of Intracellular Reactive Oxygen Species Levels

Intracellular reactive oxygen species (ROS) staining was performed with Chloromethyl 2′7′-dichlorodihydrofluorescein diacetate (H2DCFDA, #D399, Thermo Fisher), a non-fluorescent lipophilic compound that diffuses and crosses the cell membrane. Under the action of intracellular esterase, H2DCFDA deacetylates to form DCFH2, which is also non-fluorescent but is now membrane impermeable. DCFH2 then reacts with intracellular ROS (such as hydrogen peroxide) to give the fluorescent compound DCF (2′,7′-dichlorofluorescein) [56].

Briefly, cultured HepG2 or HepaRG cells were seeded in a black, clear-bottom 96-well plate at a density of 30,000 cells/well in 100 µL of basal culture medium, and incubated at 37 °C with 5% CO_2_. The following day, cells were washed with PBS, and treated with Ami (50 µM for HepG2 and 15 µM for HepaRG) or TMX (20 µM for both cells) alone or combined with MSC secretome (20 µg/mL) to a final volume of 100 µL/well and returned to the 37 °C incubator with 5% CO_2_. After 15 h of treatment, the cells were washed twice with 1× PBS. As positive control, one group of cells was treated with 500 µM H_2_O_2_ for 45 min prior to incubation with H2DCFDA.

Cells were incubated with 10 µM H2DCFDA (dissolved in culture medium without phenol red and supplementation) for 30 min at 37 °C. Fluorescence was recorded in a turner BioSystem Modulus fluorometer (ex/em 488 nm/520 nm). ROS production in the experimental groups were normalized against control cells exposed to drug solvent (0.5% DMSO).

### 2.10. In Vitro Proliferation Assay

Cell growth was first arrested by culturing HepG2 and HepaRG cells in a low-glucose medium for 24 h. The following day, cells were treated with AMI or TMX, alone or with MSC secretome. One set of plates was used to analyze the expression of genes encoding key factors related to hepatocyte proliferation as previously described, while another set was used to evaluate cell proliferation by immunofluorescence.

Briefly, cells were fixed for 15 min in 4% paraformaldehyde in PBS at RT, washed three times with PBS and incubated with anti-Ki67 antibody (ab15580; secondary antibody: anti-rabbit Alexa Fluor 555 conjugate, #4413, Cell Signaling Technology, Danvers, MA, USA); nuclei were counterstained with DAPI. Samples were analyzed by confocal microscopy. The labeling index was determined as previously described [13], counting Ki-67 (+) nuclei per 100 hepatocytes in 20 high-power fields per slide and three replicates per experimental group, using the ImageJ 1.52a software (https://imagej.nih.gov/ij/download.html, accessed on 12 November 2022).

### 2.11. Animal Model of DILI Induced by AMI

Five-week-old male C57BL/6 mice were housed at constant temperature (22 ± 2 °C) and 60% relative humidity, with 12:12-h light-dark cycle. Animals were fed with a standard diet (normal group; 10 cal% fat, 20 cal% proteins, and 70 cal% carbohydrates) or a high-fat diet (HFD) (obese group; 60 cal% fat, 20 cal% proteins, and 20 cal% carbohydrates, D12492 high-fat diet, Research Diets Inc., New Brunswick, NJ, USA), for 30 weeks. At this time, the obese group develops severe steatosis with impaired hepatic regeneration [13,58].

At this experimental point, normal and obese mice were randomly divided into three groups. The first group was treated with a weekly intra peritoneal (*i.p.*) administration of 0.9% NaCl solution (vehicle) for 4 weeks (control group). The other groups were treated with AMI 40 mg/kg (Atlansil, Pharma Investi, Santiago, Chile), *i.p.* daily for 4 weeks. At the end of each week, the second and third groups received 200 µL of 0.9% NaCl solution (AMI groups) or 200 µL of secretome derived from 2 × 10^6^ MSCs (MSC secr groups), respectively, by tail vein. Animals were sacrificed 72 h after the last vehicle or MSC secretome administration. Blood samples were collected and plasma stored at −80 °C until use. The hepatic tissue was preserved for subsequent molecular and histological analyses. All animal protocols were approved by the CICUAL Committee of Universidad del Desarrollo.

### 2.12. Biochemical Analyses

Lipid profile, blood glucose, serum alanine aminotransferase (ALT), and aspartate aminotransferase (AST) were determined by automatized methods how was previously described [58].

### 2.13. Liver Histology and Immunofluorescence Analyses

For histological analyses, serial 4 µm sections of the right lobe of the livers were stained with hematoxylin and eosin to evaluate hepatic steatosis and leukocyte infiltration, or with Masson’s trichrome stain to evaluate liver fibrosis.

A standardized score including steatosis, inflammatory foci, and hepatocyte ballooning was employed to evaluate NAFLD [59,60].

Confocal microscopy was used to count liver infiltrating macrophages (F4/80+) and T lymphocytes (CD3+), and to evaluate liver fibrogenesis (α-SMA immunoreactivity). Sections were blocked with 5% FBS in Tris-buffered saline (TBS) and incubated with the primary antibodies F4/80 ab74383, α-SMA ab5694 (Abcam, Fremont, CA, USA), or CD3 A0452 (Dako, Santa Clara, CA, USA) in Signal Stain diluent (Cell Signaling Technology) at 4 °C overnight. After washing, sections were incubated with secondary antibody at RT for 1 h and nuclei were counterstained with DAPI. Sections were examined with the Fluoview FV101 confocal microscope (Olympus, Shinjuku, Japan).

The CD3+ and F4/80+ cells in hepatic tissue were assessed semiquantitatively in 15 random sections per animal and expressed as number of positive cells per tissue section [60].

To determine the hepatic proliferation rate, 4 µm sections of the right lobe of the livers were stained for proliferating cell nuclear antigen (PCNA) using the anti-PCNA NB600-1331 antibody (Novus Biologicals, Centennial, CO, USA) and evaluated by confocal microscopy as previously described [13]. Hepatic apoptosis was assessed by the terminal deoxynucleotidyl transferase-mediated dUTP nick end labeling (TUNEL) method using the DeadEnd™ Fluorometric TUNEL System (Promega, USA). Nuclei were counterstained with DAPI and fluorescence was evaluated by confocal microscopy (Fluoview FV10i, Olympus). Labeling indices were determined counting PCNA (+) and TUNEL (+) nuclei per 100 hepatocytes in 30 high-power fields per liver and six livers per experimental group; the ImageJ 1.52a software was used for cell quantification.

### 2.14. Proteomic Analysis of the Secretome of MSCs Obtained from Human Adipose Tissue

The protein composition of the secretome of adipose tissue MSCs obtained from three donors was analyzed by nanoLC-MS/MS on a Q-Exactive “classic” mass spectrometer (Thermo Fisher Scientific) in data-dependent mode how was previously described [61].

Three technical replicates were acquired for each biological replicate. Data were processed in the MaxQuant/Perseus software suite (v.1.6.2) (https://maxquant.net/perseus/, accessed on 12 November 2022) for protein identification and label-free quantification. Data were subjected to ontology and pathway analyses using the protein analysis through evolutionary relationship tool (PANTHER classification system) and gene ontology algorithms, and were classified based on pathways, biological processes, and molecular functions.

### 2.15. Statistical Analyses

Data are presented as means ± SEM. To analyze the statistical significance of intergroup differences, the Kruskal–Wallis test was used to compare mean values among all groups, and the Mann–Whitney U test was used to compare mean values between two groups. *p* < 0.05 was considered statistically significant.

## 3. Results

### 3.1. MSC Secretome Increased Cell Viability on Amiodarone or Tamoxifen Injured Hepatocytes

To study if the MSC secretome confers a hepatoprotective effect in DILI, AMI and TMX were used to establish in vitro models of DILI in two representative hepatocyte cell lines, HepG2 and HepaRG. First, as control, several MSC secretome concentrations were tested on both cell lines for 24 h to assess its possible cytotoxicity. Both cell lines maintained their viability when incubated at different secretome concentrations (from 20 to 100 µg/mL), suggesting that MSC secretome is not cytotoxic (Figure S1).

The cytotoxic effects of AMI and TMX were measured by MTT assay on HepG2 and HepaRG cells after 24 h of incubation. As shown in Figure S2, cell viability decreased significantly in a dose-dependent manner in both cell types. HepaRG cells were more sensitive to both drugs than HepG2 cells. For the next experiments, two drug concentrations were chosen to evaluate the cytoprotective effect of MSC secretome (20 and 50 µM AMI; 15 and 20 µM TMX for HepG2 cells, 10 and 15 µM AMI; 15 and 20 µM TMX for HepaRG cells).

AMI has two main metabolites, mono-N-desthylamiodarone (MDEA) and di-desthylamiodarone (DDEA), which are at least partially responsible for its hepatocellular toxicity [62]. The initial steps of AMI metabolism are catalyzed predominantly by the cytochrome P450 (CYP) enzyme CYP3A4 [63], but also by CYP 1A1, 1A2, 2C9, 219, and 2D6 [64]. Similarly, TMX is a prodrug that is metabolized to its active metabolite (4-hydroxy-tamoxifen) by CYP 2B6, 2D6, 3A4, and 3A7 [65].

The basal mRNA levels of drug-metabolizing CYP450 enzymes were measured in HepG2 and HepaRG cells. In accordance with previous reports and with the increased HepaRG sensitivity observed in this study, the mRNA levels of the most relevant CYP for AMI and TMX metabolism (3A4, 2D6, 1A1, 1A2, 2C9, and 2C19) [66] were significantly higher in HepaRG cells than in HepG2 cells (Figure S3).

The hepatoprotective effect of the MSC secretome was determined by coincubating HepG2 or HepaRG cells with MSC secretome and AMI or TMX for 24 h. Two different end points, mitochondrial activity in viable cells (MTT assay) and plasma membrane integrity (LDH release assay), were used to measure the general cytoprotective effect of the MSC secretome.

**Figure 1:**
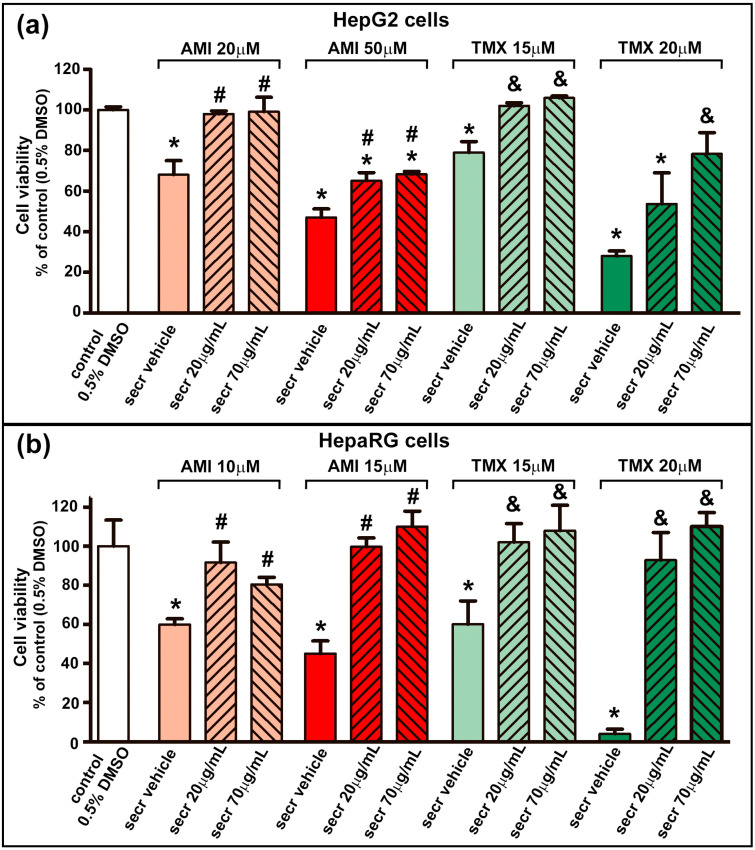
MSC secretome presented a cytoprotective effect in hepatic cells incubated with AMI or TMX. To determine the cytoprotective effect of MSC secretome on (**a**) HepG2 and (**b**) HepaRG cells, both cell lines were incubated with AMI (light and dark red bars) or TMX (light and dark green bars) at the indicated concentrations, alone or coincubated with two concentrations of MSC secretome (20 or 70 µg/mL—line pattern bars), or the secretome vehicle (PBS) for 24 h. As a control group, cells were incubated with the drug solvent (0.5% DMSO—white bars). Cell viability was measured by an MTT assay at the end of the treatment, and control group value was obtained as 100%. AMI and TMX significantly decreased cell viability in both cell types in a concentration-dependent manner, while coincubation with MSC secretome (both concentrations) led to increased cell viability in both cell lines. Data are presented as means ± SEM (*n* = 4) of three independent experiments. * *p* < 0.05 vs. control group (0.5% DMSO); # *p* < 0.05 vs. AMI plus secretome vehicle, of the same experimental group (same AMI concentration); & *p* < 0.05 vs. TMX plus secretome vehicle, of the same experimental group (same TMX concentration). secr vehicle: MSC secretome vehicle (PBS); secr: MSC secretome; AMI: amiodarone; TMX: tamoxifen.

As shown in Figure 1, AMI and TMX significantly decreased cell viability in both cell types in a concentration-dependent manner when measured by the MTT assay, while coincubation with MSC secretome (20 or 70 µg/mL) led to increased cell viability in both DILI in vitro models (HepG2 and HepaRG cells). With these results, for the following experiments, the lowest MSC secretome concentration was evaluated.

**Figure 2:**
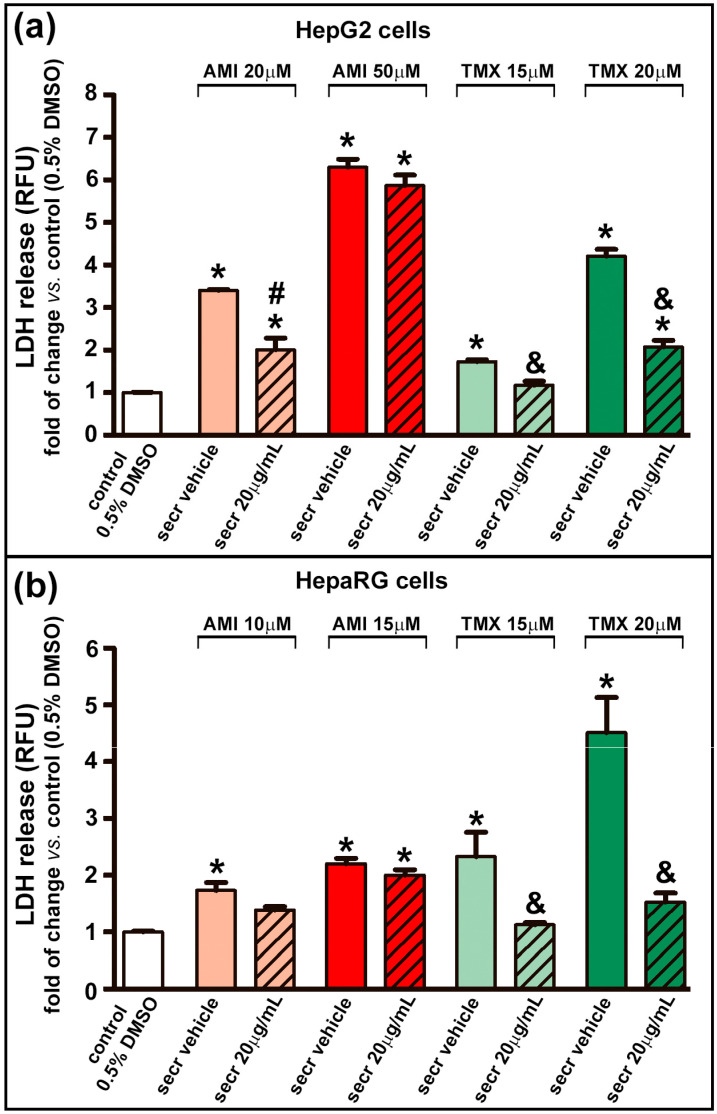
MSC secretome decreased the release of LDH in hepatic cells incubated with TMX. The integrity of the plasma membrane was evaluated by the release of LDH into the culture medium in (**a**) HepG2 and (**b**) HepaRG cells exposed to AMI (light and dark red bars) or TMX (light and dark green bars) alone or coincubated with MSC secretome (20 µg/mL—line pattern bars), or the secretome vehicle (PBS) for 24 h. As a control group, cells were incubated with the drug solvent (0.5% DMSO—white bars). The amount of LDH released in the culture medium was determined by a fluorescent method (shown as relative fluorescence units), and expressed as fold of change vs. control group (0.5% DMSO). A dose-dependent release of LDH was observed in both cell lines when exposed to AMI or TMX. While a moderate effect was seen in HepG2 cells treated with 20 µM AMI plus secretome. Data are presented as means ± SEM (*n* = 4) of three independent experiments. * *p* < 0.05 vs. control group (0.5% DMSO); # *p* < 0.05 vs. AMI plus secretome vehicle, of the same experimental group (same AMI concentration); & *p* < 0.05 vs. TMX plus secretome vehicle, of the same experimental group (same TMX concentration). secr vehicle: MSC secretome vehicle (PBS); secr: MSC secretome; AMI: amiodarone; TMX: tamoxifen. LDH: lactate dehydrogenase. RFU: relative fluorescence units.

Cell necrosis was assessed by measuring the leakage of LDH into the culture medium as an indicator of the loss of integrity of the plasma membrane. A dose-dependent release of LDH was observed in both cell lines when exposed to AMI or TMX. As shown in Figure 2, the coincubation with MSC secretome provided a cytoprotective effect against TMX in both cell lines (evidenced by a decrease in the LDH release). However, only a moderate effect was seen in HepG2 cells treated with 20 µM AMI plus MSC secretome at 20 µg/mL.

Collectively, these results suggest that there are some factors in the MSC secretome which are involved in the protection of hepatic cells from DILI mediated by AMI or TMX.

### 3.2. MSC Secretome Prevents Cytochrome Release from Mitochondria and Protects Hepatocytes from Apoptosis by Decreasing Caspase 3/7 Activity

Cytochrome C is normally located in the space between the inner and the outer mitochondrial membranes. The AMI and TMX cytotoxic effects have been related to mitochondrial membrane disruption, leading to a release of cytochrome C into the cytoplasm, and apoptosis induction [67]. Therefore, we evaluated mitochondrial cytochrome C release by confocal microscopy as previously described by Zahno et al. [56]. Mitochondria were labeled with a fluorescent MitoTracker reagent, while cytochrome C with a monoclonal antibody was conjugated with Alexa Fluor 488 (Cell Signaling, Danvers, MA, USA).

**Figure 3:**
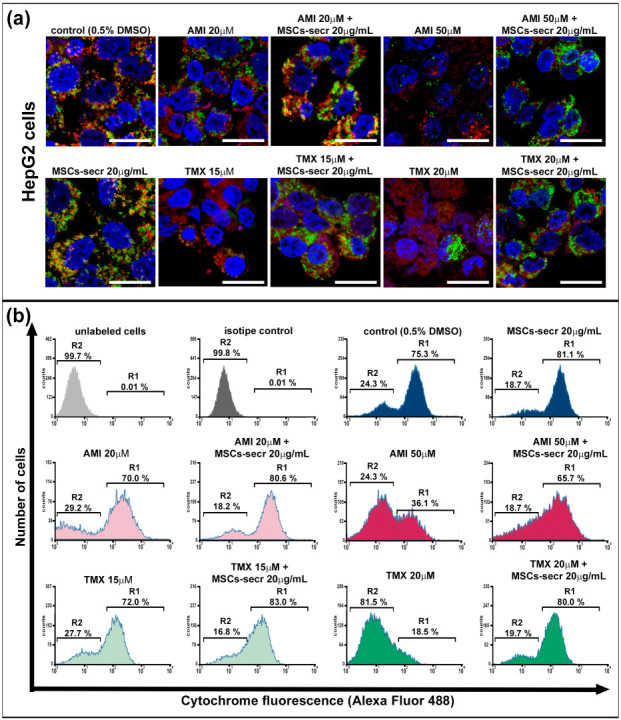

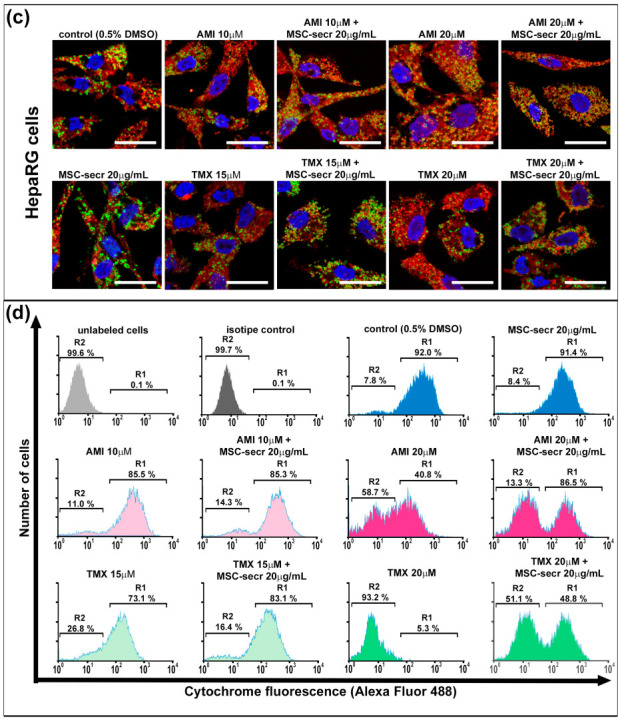
Coincubation with MSC secretome prevented cytochrome C release in HepG2 and HepaRG cells exposed to AMI or TMX. The release of cytochrome C was evaluated in HepG2 and HepaRG cells exposed to AMI or TMX alone or coincubated with MSC secretome (20 µg/mL) for 24 h. Mitochondrial cytochrome C release was detected by immunofluorescence in (**a**) HepG2 cells and (**c**) HepaRG cells. Representative micrographs of mitochondria (MitoTracker, red), cytochrome C (Cyt C, green), and their colocalization (merge, yellow); nuclei were counterstained with DAPI (blue). Cells with drug solvent (0.5% DMSO) were used as control group and showed maximal colocalization. Scale bars represent 20 µm. AMI and TMX induced mitochondrial damage with the concomitant release of cytochrome C in both cell lines, particularly at higher concentrations (evidenced by the loss of colocalization). On the other hand, control groups and cells coincubated with AMI or TMX plus MSC secretome exhibited colocalization of mitochondria and cytochrome C. Semiquantitative determination of cytochrome C release was evaluated by flow cytometry. Fluorescence histograms of immunolabeled cytochrome C in (**b**) HepG2 and (**d**) HepaRG cells. The cells were incubated with AMI or TMX alone or coincubated with MSC secretome (20 µg/mL) for 24 h, permeabilized with digitonin and labeled with anti-cytochrome C Alexa Fluor 488 antibody. Unlabeled cells (autofluorescence), and cells incubated with isotype antibody (isotype control), were used as negative control, whereas cells with drug solvent (0.5% DMSO) were used as control group and showed maximal cytochrome C fluorescence. Region 1 (R1) and region 2 (R2), were arbitrary defined to limit the population of cells with high and low fluorescence, respectively. Numbers at top of R1 and R2 regions are percentages of cells in each region. Control cells (0.5% DMSO) and cells incubated with MSC secretome, presented the majority of the cells in R1 (cells that have not yet released their mitochondrial cytochrome C), whereas cells exposed to the highest AMI or TMX concentrations showed a decrease in the cells in R2 (cells that have already released their mitochondrial cytochrome C). Remarkably, the MSC secretome coincubation prevented this transition. Histograms are representative of four independent experiments, presented in Figure S5.

As shown in Figure 3a,c and Figure S4, exposure to AMI and TMX induced mitochondrial damage with the concomitant release of cytochrome C in both cell lines, especially at higher drug concentrations, as evidenced by loss of colocalization of mitochondria (red) and cytochrome C (green) in the micrographs. In contrast, control groups and cells coincubated with AMI or TMX plus MSC secretome exhibited the colocalization of mitochondria and cytochrome C.

To complement these observations, a semiquantitative determination of cytochrome C release during cell death by flow cytometry analysis of digitonin-permeabilized hepatic cells immunolabeled for cytochrome C (Alexa Fluor 488) was performed [57,68]. (Figure 3b,d). The cells were cultured in each condition for 24 h, and we determined the fraction of cells that have not yet released their mitochondrial cytochrome C and were still highly fluorescent, as well the fraction of apoptotic cells that have already released their mitochondrial cytochrome C and therefore, were not fluorescent. Region 1 (R1) and region 2 (R2), were arbitrarily defined to limit the population of cells with high and low fluorescence, respectively.

Cells not labeled with any antibody were used as the autofluorescence control (light gray histogram), whereas cells incubated with the isotype antibody were used as the isotype control (dark gray histogram). Both groups of cells were located in R2.

Confirming the results obtained by microscopy (Figure 3a,c and Figure S4), control cells incubated with the drug diluent (0.5% DMSO) and cells incubated with MSC secretome alone presented the majority of the cells in the region of high fluorescence R1.

On the other hand, cells undergoing apoptosis induced by AMI or TMX show a dose-dependent decrease in the high fluorescent cell population, with a concomitant increase in the low fluorescent cell population in R2. Remarkably, the MSC secretome coincubation prevented this transition (Figure 3b,d and Figure S5).

**Figure 4:**
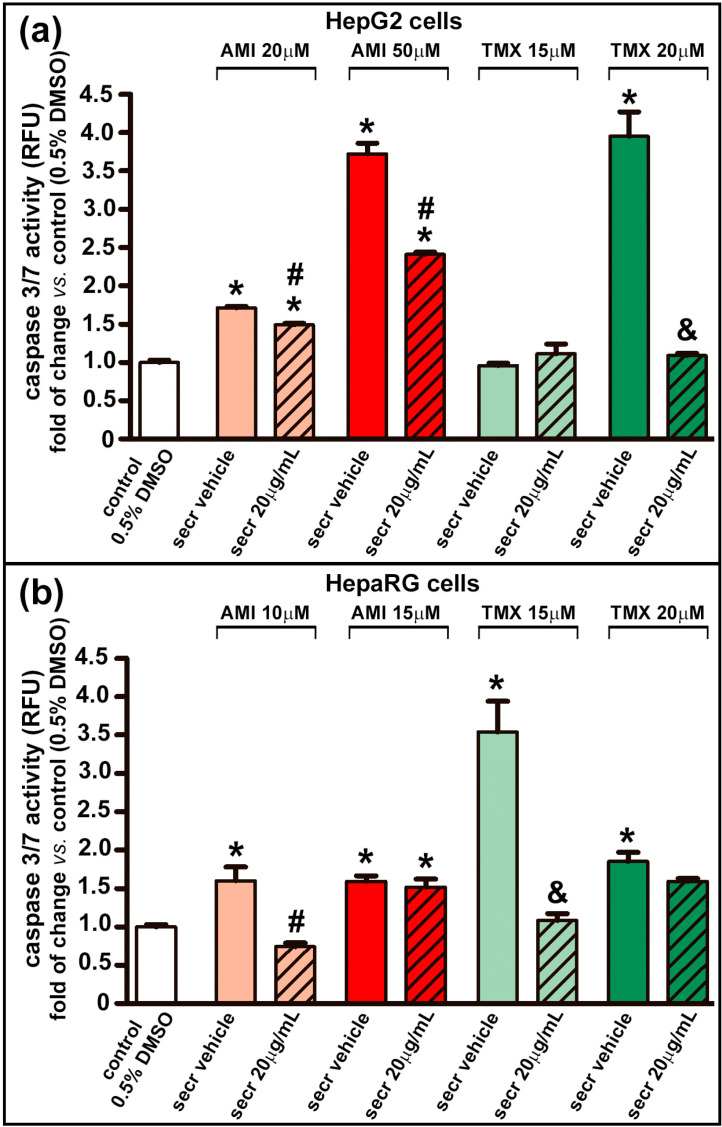
MSC secretome decreased caspase 3/7 activity in hepatic cells incubated with AMI or TMX. The activity of caspase 3/7 was evaluated in (**a**) HepG2 and (**b**) HepaRG cells exposed to AMI (light and dark red bars) or TMX (light and dark green bars) alone or coincubated with MSC secretome (20 µg/mL—line pattern bars), or the secretome vehicle (PBS) for 24 h. As a control group, cells were incubated with the drug solvent (0.5% DMSO—white bars). The activity of caspase 3/7 was evaluated by a fluorescent method (relative fluorescence units), and expressed as fold of change vs. control group (0.5% DMSO). The activity of caspase 3/7 was increased in both cell types after treatment with AMI or TMX (20 µM for HepG2 cells and 15 or 20 µM for HepaRG cells), whereas treatment with MSC secretome suppressed the activity of caspase 3/7. Data are presented as means ± SEM (*n* = 4) of three independent experiments. * *p* < 0.05 vs. control group (0.5% DMSO); # *p* < 0.05 vs. AMI plus secretome vehicle, of the same experimental group (same AMI concentration); & *p* < 0.05 vs. TMX plus secretome vehicle, of the same experimental group (same TMX concentration). secr vehicle: MSC secretome vehicle (PBS); secr: MSC secretome; AMI: amiodarone; TMX: tamoxifen. RFU: relative fluorescence units.

The role of cytochrome C release in promoting caspase activation is widely accepted [69]. The activity of caspase 3/7, a key mediator of apoptosis, was increased in both cell types after treatment with AMI for 24 h, and with TMX (20 µM for HepG2 cells and 15 or 20 µM for HepaRG cells) (Figure 4). Similarly to the favorable outcome seen in cell viability in the cell model of injury by AMI, treatment with MSC secretome significantly suppressed the activity of caspase 3/7. In this results, the most interesting data came from drug concentrations that induced greater caspase 3/7activity (AMI 50 µM and TMX 20 µM for HepG2; and TMX 15 µM for HepaTG).

Interestingly, the caspase 3/7 activity of HepaRG cells treated with 20 µM TMX was lower than that of the group treated with 15 µM TMX. This result may be attributed to the necrosis effect induced by this concentration of TMX (shown in Figure 2a and Figure S6).

### 3.3. MSC Secretome Decrease Intracellular Reactive Oxygen Species Production in Hepatic Cells

**Figure 5:**
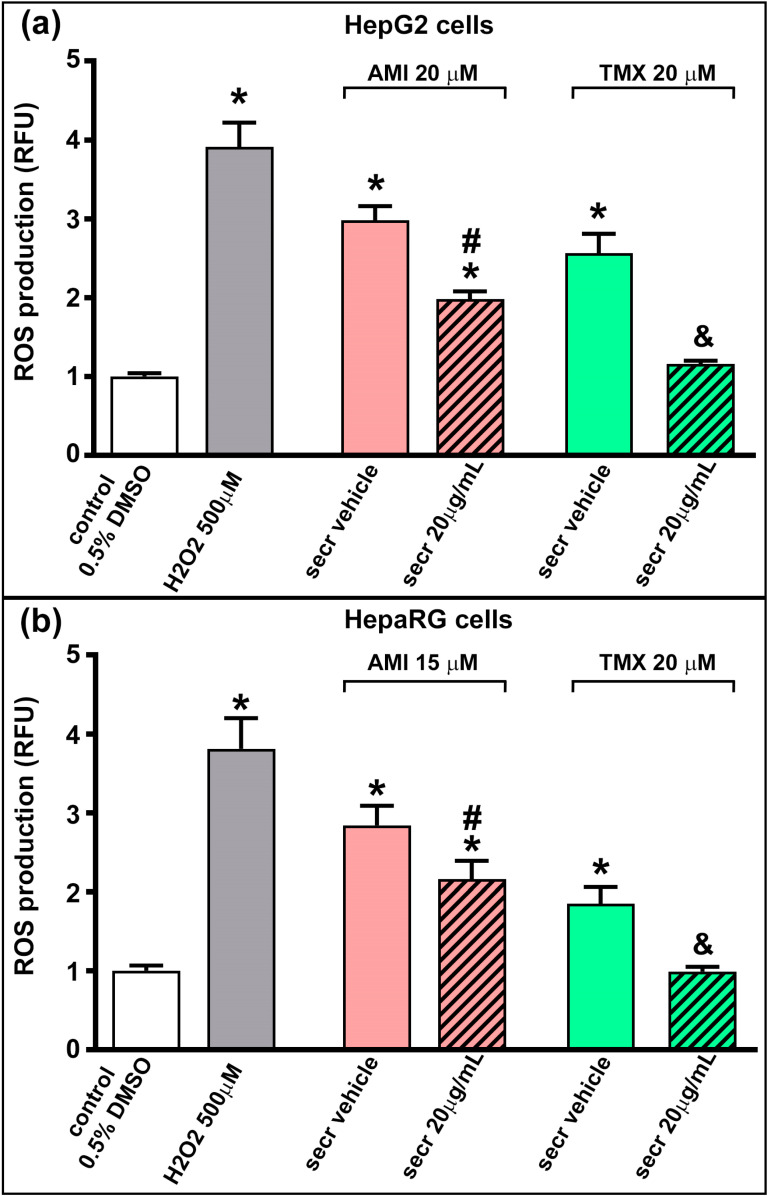
MSC secretome decreased intracellular ROS production in hepatic cells incubated with AMI or TMX. The production of reactive oxygen species (ROS) was evaluated in (**a**) HepG2 and (**b**) HepaRG cells exposed to AMI (red bars) or TMX (green bars) alone or coincubated with MSC secretome (20 µg/mL—line pattern bars) for 15 h. As control group, cells were incubated with the drug vehicle alone (0.5% DMSO—white bars), whereas peroxide hydrogen (H_2_O_2_) was used as positive control (gray bars). Intracellular ROS staining was performed with H2DCFDA (10 µM), and the fluorescence intensity was quantified and expressed as fold of change vs. control group (0.5% DMSO). The exposure to AMI and TMX induced ROS production in both cell lines (evidenced by the increased fluorescence), whereas the coincubation with MSC secretome reduced the ROS production. Data are presented as mean ± SEM (*n* = 6) of three independent experiments. * *p* < 0.05 vs. control group (0.5% DMSO); # *p* < 0.05 vs. AMI plus secretome vehicle (PBS) of the same experimental group; & *p* < 0.05 vs. TMX plus secretome vehicle (PBS) of the same experimental group. RFU: relative fluorescence units; secr vehicle: (secretome vehicle, PBS); secr: (MSC secretome).

ROS formation can be related to the inhibition of the electron transport chain, which has been demonstrated in hepatocytes, for AMI and TMX [23]. We therefore evaluated whether the coincubation with MSC secretome could reduce the ROS production in HepG2 and HepRG cells. As shown in Figure 5, exposure to AMI and TMX induced ROS production in both cell lines, as evidenced by the increased fluorescence. In line with the above results, treatment with MSC secretome (20 µg/mL) significantly reduced the ROS production.

### 3.4. MSC Secretome Induces Proliferation in HepaRG Cells

The hepatic regenerative response is based in the hepatocyte replication, which is regulated by a complex interaction of paracrine and endocrine signals. Previous reports have shown that both AMI and TMX reduce the proliferation of hepatic cells in a concentration- and time-dependent manner [55,70]. To determine whether the MSC secretome can directly enhance hepatocyte replication, we evaluated its effect on the proliferation rate of HepG2 and HepaRG cells. The cell growth was arrested by culturing cells in a low glucose medium for 24 h, and the treatment with AMI or TMX was introduced either with or without MSC secretome (20 and 70 µg/mL were studied), whereas the cell proliferation was evaluated after 24 h of treatment.

**Figure 6:**
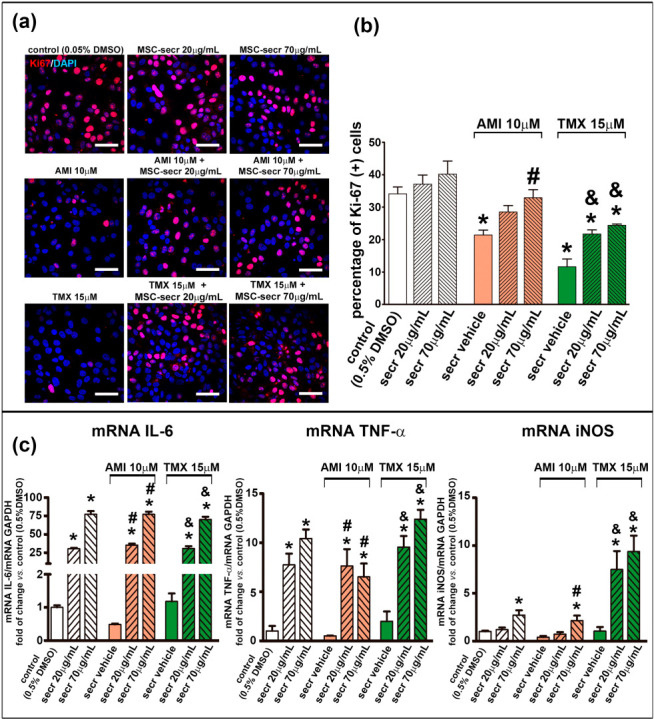
Coincubation with MSC secretome enhanced HepaRG proliferation after exposure to AMI or TMX. Cell proliferation was evaluated in HepRG cells exposed to AMI (red bars) or TMX (green bars) alone or coincubated with two concentrations of MSC secretome (20 and 70 µg/mL—line pattern bars) for 24 h. As a control group, cells were incubated with the drug solvent (0.5% DMSO—white bars). Ki-67 immunoreactivity (Alexa Fluor 555, red) was evaluated by immunofluorescence. Nuclei were counterstained with DAPI (blue). The proliferation rate decreased in the HepaRG cells, when were exposed to AMI or TMX. However, coincubation with MSC secretome stimulated the proliferation of the cells in both conditions (AMI and TMX exposure). (**a**) Representative micrographs of HepaRG cells. Scale bars represent 50 µm. (**b**) Quantification of Ki-67-positive nuclei was carried out by digital image analysis. All data are presented as means ± SEM of Ki-67-positive nuclei per 100 hepatocytes in 20 high-power fields per slides and three replicates per experimental group. (**c**) To complement the evaluation, the expression of key factors in the proliferative response after 24 h of treatment, was evaluated by RT-qPCR, normalized against GAPDH, and expressed as fold of change vs. control group (0.5% DMSO). In line with the proliferation rate, the mRNA levels of IL-6, TNF-α and iNOS were increased in the groups incubated with MSC secretome. Data are presented as means ± SEM (*n* = 4) of three independent experiments. * *p* < 0.05 vs. control group (0.5% DMSO); # *p* < 0.05 vs. AMI plus secretome vehicle, of the same experimental group (same AMI concentration); & *p* < 0.05 vs. TMX plus secretome vehicle, of the same experimental group (same TMX concentration). secr vehicle: MSC secretome vehicle (PBS); secr: MSC secretome; AMI: amiodarone; TMX: tamoxifen.

The number of cells presenting the proliferation marker Ki-67 was counted by confocal microscopy to estimate proliferation rates. As shown in Figure 6a,b and Figure S7, the proliferation rate decreased in both cell lines when exposed to AMI or TMX. However, coincubation with MSC secretome stimulated the proliferation of HepaRG cells in both conditions (AMI and TMX exposure).

Both IL-6 and TNF-α are key factors in initiating liver regeneration on injury by committing cell cycling, whereas iNOS mediate the formation of nitric oxide (NO) which catalyzes the synthesis of prostaglandins that control the regenerative process [71,72,73]. To complement the evaluation of the proliferative response, the expression of IL-6, TNF-α, and iNOS was determined by RT-qPCR. In agreement with the proliferation rate, the mRNA levels of IL-6, TNF-α, and iNOS in HepaRG were increased in the groups coincubated with MSC secretome (Figure 6c). In contrast, expression of these genes was not stimulated in HepG2 cells (Figure S7).

### 3.5. In Vivo Administration of MSC Secretome Improves Liver Histopathology and Reduces the Local Inflammatory Process

To strengthen our results from in vitro studies, as proof of concept, we studied the effect of MSC secretome administration in an animal model of DILI induced by the chronic administration of AMI. Mice were fed with a control diet or with HFD to induce hepatic steatosis, a very frequent condition associated with DILI in humans and characterized by the inhibition of the endogenous capacity of the liver to regenerate [7,13].

The livers from mice fed with a control diet and exposed to a daily dose of AMI (40 mg/kg/day for four weeks) exhibited no signs of pathology, and no effects on biochemical parameters or body weight. Additionally, the liver histology of lean mice treated with AMI was not different from control mice treated with vehicle, and both groups were negative for inflammation, necrosis, or steatosis (Figure S8).

At 30 weeks, the body weight of mice fed with HFD almost doubled that from mice fed with a standard control diet. Serum cholesterol, blood glucose, and plasma insulin levels were increased in obese mice (Figure S9).

**Figure 7:**
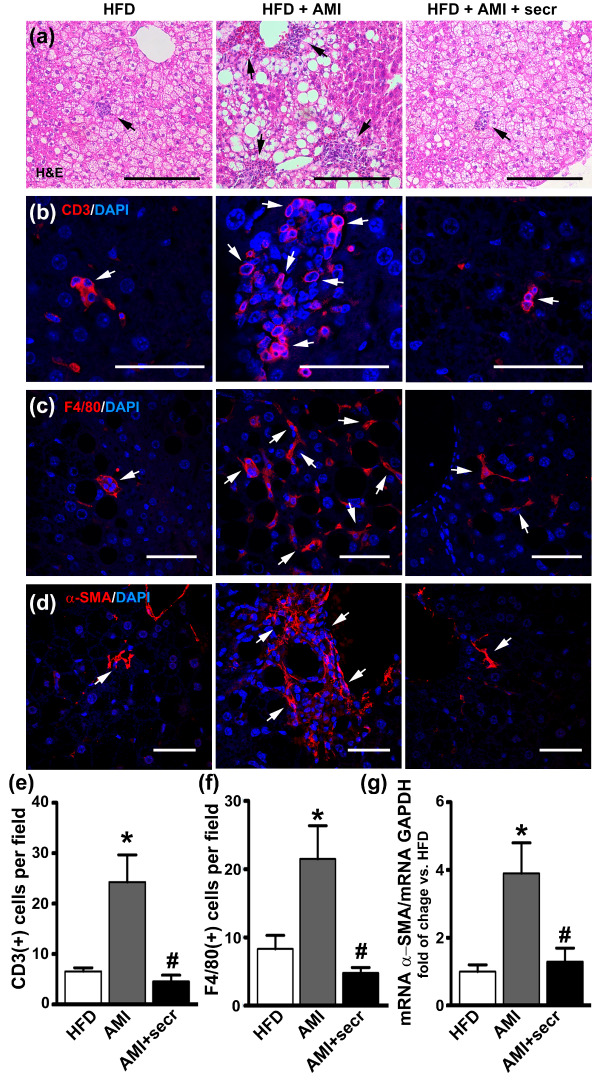
MSC secretome administration prevented hepatic injury induced by AMI in an obese mouse model. Male mice were fed a high-fat diet (HFD) for 34 weeks and divided into three groups. During the last four weeks, one group did not receive additional treatment (HFD group), while a second group was treated daily with AMI (40 mg/kg) (HFD + AMI). The third group received MSC secretome endovenously once a week (HFD + AMI + secr). The microscopic evaluation of liver sections of obese mice treated with AMI revealed predominance of macrosteatosis, profound hepatocellular death with cytoplasmatic vacuolization, severe distortion of tissue architecture and multifocal inflammatory cellular foci. On the other hand, the coadministration of AMI with MSC secretome to obese animals resulted in great improvement of the histological appearance of the hepatic tissue, with predominance of microsteatosis and no evidence of inflammatory response. Additionally, a decrease in the number of infiltrating T lymphocytes macrophages cells in animals treated with AMI plus MSC secretome is observed. Representative micrographs of liver sections are shown. (**a**) Hematoxylin and eosin staining. The presence of inflammatory foci is indicated by arrows (scale bars represent 200 µm). Infiltration of T lymphocytes by (**b**) CD3 (Alexa 555, red) and (**c**) macrophages by F4/80 (Alexa 555, red) was evaluated by immunofluorescence. Nuclei were counterstained with DAPI (blue). Quantification of CD3 (**e**) and F4/80 (**f**) positive cells (arrows) was carried out by digital image analysis. The data are presented as means ± SEM of 30 random fields per animal and six animals per group. * *p* < 0.05 vs. control HFD mice; # *p* < 0.05 vs. HFD + AMI mice. To study the hepatic fibrosis, the immunoreactivity of α-SMA (**d**) was determined by confocal microscopy (Alexa 555, red) and the α-SMA mRNA levels (**g**) were measured by RT-qPCR. Alpha-SMA staining was almost completely absent from samples of untreated obese mice, however marked α-SMA immunoreactivity appear localized in perisinusoidal and pericellular areas of the livers from AMI-treated mice, while minimal staining was observed in animals treated with secretome. This result is in line with the hepatic level of α-SMA. Gene expression was normalized against GAPDH and expressed as fold of change vs. HFD-control group. Data are presented as means ± SEM (*n* = 4). * *p* < 0.05 vs. HFD-control group; # *p* < 0.05 vs. HFD + AMI.

Compared with mice kept on a standard control diet, mice fed with the HFD developed severe hepatic steatosis (Figure 7a, left panel), which has been associated with increased content of triglycerides in the liver [13,48].

The microscopic evaluation of the H&E-stained liver sections of the obese mice treated with AMI revealed a predominance of macrosteatosis, profound hepatocellular death with cytoplasmic vacuolization, severe distortion of tissue architecture, and multifocal inflammatory cellular foci (Figure 7a, middle panel). In contrast, the coadministration of AMI with MSC secretome to obese animals resulted in great improvement in the histological appearance of the hepatic tissue, with a predominance of microsteatosis and no evidence of inflammatory response (Figure 7a, right panel, and Figure S10). In addition, semiquantitative histological examination of the hepatic tissue by confocal microscopy confirmed a decrease in the number of infiltrating T lymphocytes (CD3+) and macrophage (F4/80+) cells in animals treated with AMI plus MSC secretome (Figure 7b,c,e,f).

No collagen deposition was observed in the liver parenchyma in obese animals of any experimental group (Masson’s trichrome, Figure S9). However, at the initial stages of liver fibrosis, chronic hepatocyte injury induces the activation of HSCs into myofibroblast-like cells with an increased expression of α-SMA and triggers the hepatic fibrosis [74]. Immunofluorescence staining was performed for α-SMA to evaluate HSCs activation after AMI administration. As shown in Figure 7d, staining was almost completely absent from samples of untreated obese mice. However, marked staining of α-SMA appeared to be localized in perisinusoidal and pericellular areas of the livers from AMI-treated mice, although minimal staining was observed in animals treated with secretome. In the same line, semiquantitative analysis of α-SMA mRNA demonstrated higher levels in AMI-treated mice than in the control and MSC secretome-treated groups (Figure 7g).

Collectively, these results demonstrate that MSC secretome administration inhibits the development of histopathological changes and immune cell infiltration in liver from obese mice treated with AMI.

### 3.6. In Vivo Administration of MSC Secretome Improves Liver Regeneration

**Figure 8:**
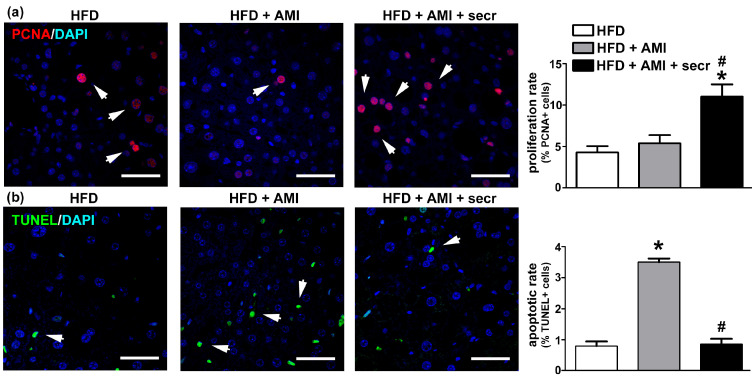
MSC secretome administration enhanced proliferation and inhibited apoptosis of hepatic cells after chronic exposure to AMI. Cell proliferation and apoptosis were analyzed in all experimental groups 24 h after the last administration of AMI. The effect of MSC secretome (secr) on cell proliferation was evaluated by PCNA immunoreactivity (Alexa Fluor 555, red), while cell apoptosis was determined by TUNEL staining (FITC, green). In both cases, nuclei were counterstained with DAPI (blue). Few PCNA (+) cells were seen in steatotic livers, regardless of whether the mice had been treated chronically with vehicle (control) or AMI, whereas in the group treated with MSC secretome significantly more PCNA (+) nuclei were observed. On the other hand, mice in the obese group exposed to AMI presented an increased basal apoptotic rate, whereas a significant reduction in TUNEL (+) nuclei was observed when MSC secretome was administered jointly with AMI. Representative micrographs of liver tissue in which hepatocyte proliferation was determined by (**a**) PCNA labeling or (**b**) TUNEL are shown (arrows). Scale bars represent 50 µm. Quantification of PCNA- and TUNEL-positive nuclei was carried out by digital image analysis. All data are presented as means ± SEM for 30 random fields per animal and six animals per group. * *p* < 0.05 vs. control HFD mice; # *p* < 0.05 vs. HFD + AMI mice.

Stimulation of the endogenous repair program represents another potential mechanism associated with the therapeutic effects induced by MSC secretome. To determine the hepatic proliferative activity, immunofluorescence staining for the proliferation marker PCNA was performed in obese mice. As shown in Figure 8a, quantitatively few PCNA (+) cells were seen in steatotic livers, regardless of whether the mice had been treated chronically with vehicle (control) or AMI, whereas in the group treated with MSC secretome, significantly more PCNA (+) nuclei were observed.

Regarding the type of hepatic cells in proliferation, reports have demonstrated that hepatocytes are the principal proliferating cells [13]. In this study, the identity of hepatocytes as the principal proliferating cells was confirmed by the characteristic shape of the nuclei (distinctly round, with one or two prominent nucleoli), and a double immunofluorescence signal for albumin and PCNA (Figure S11a).

On the other hand, mice in the obese group exposed to AMI presented an increased basal apoptotic rate, whereas a significant reduction in TUNEL (+) nuclei was observed when MSC secretome was administered jointly with the drug (Figure 8b and Figure S12). Although, the identity of apoptotic cells is more difficult to determine because cells are irregularly shaped and have lost many of their markers in the late apoptotic process detected by TUNEL (Figure S11b). However, the literature supports the concept that the hepatocyte is the principal apoptotic cell observed in DILI [69].

The above results confirm the increased susceptibility of the steatotic liver to DILI and show that systemic administration of MSC secretome in the studied animal model reduces the pathological mechanism by both inducing hepatocyte proliferation and reducing hepatocellular death.

### 3.7. Proteomic Analysis of the Secretome of MSCs Obtained from Human Adipose Tissue

The MSC secretome is known to contain several molecules, including—but not limited to—growth factors, hormones, metabolites, polysaccharides, and proteins [75]. We performed an LC-MS/MS analysis to identify secreted proteins contained in the MSC secretome used to treat the DILI phenotype in vitro and in vivo.

**Figure 9:**
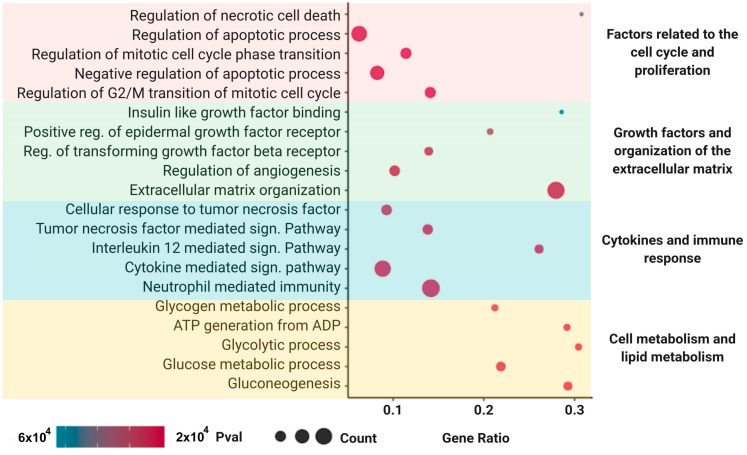
Mass spectrometry and enrichment analysis of the MSC secretome derived from adipose human MSCs. Functional enrichment analysis (*p* < 0.05) of the differentially expressed proteins according to the Reactome, Gene Ontology (Molecular Function and Biological Process), KEGG, and Wiki Pathways databases. Enriched functional categories were chosen on the basis of their association with the hepatic regenerative process described in this work. A total of 570 proteins were identified in the MSC secretome. The analysis revealed enrichment in proteins associated with cell cycle control and proliferation, growth factors, and organization of the extracellular matrix, as well to the immune response and lipid metabolism.

A total of 570 proteins were identified in the MSC secretome. In order to obtain additional information of their possible functional roles, we performed an overrepresentation enrichment analysis using several databases related to function, pathways, and biological processes. The analysis revealed enrichment in proteins associated with cell cycle control and proliferation, growth factors, and organization of the extracellular matrix, as well as to the immune response and lipid metabolism, which are closely related to the molecular pathways involved in liver regeneration after injury (Figure 9). The detailed list of proteins identified in the MSC secretome is provided in Table S2.

## 4. Discussion

DILI is defined as the hepatic insult caused by the intake of prescription and nonprescription drugs, ranging from asymptomatic to fulminant hepatic failure, leading to life-threatening complications [76]. It is currently the single most common cause of ALF in developed countries and, furthermore, it is estimated that 10–20% of ALF cases of unknown etiology are related to DILI [77,78].

Multiple drugs related to DILI, either directly or through the effect of their metabolites, can induce mitochondrial dysfunction. Both the impairment of β-oxidation, resulting in the accumulation of unmetabolized free fatty acids (FFAs), and the lack of aerobic respiration induce an excessive production of reactive oxygen species (ROS) which ultimately leads to activation of the mitochondrial permeability transition pore, a decrease in the levels of ATP, and activation of the caspase pathway by the release of cytochrome C from mitochondria [67,79].

Although apoptosis has been perceived to be a noninflammatory process, an immune response can be initiated by the release of damage-associated molecular patterns (DAMPs) from injured cells, and their recognition by toll-like receptors on immune cells, mainly by resident Kupffer cells [80], inducing the release of cytokines and recruiting neutrophils and macrophages derived from monocytes into injured areas, which in turn induce HSC activation and liver fibrogenesis [81,82,83], thus perpetuating a vicious cycle of positive feedback. To overcome this problem, it is necessary to develop safe and efficient therapeutic alternatives to prevent further damage to the injured liver, decrease the inflammatory response, and stimulate the remaining hepatic cells to regenerate and restore liver function avoiding long-term sequelae.

Diverse evidence indicates that the administration of MSCs can improve hepatic function in different pathological conditions. However, the current consensus indicates that transplanted MSCs do not survive long, so the therapeutic effect associated with their administration is mediated, at least in part, by the secretion of their secretome, without requiring hepatic engraftment [18,84,85].

The MSC secretome contains both microvesicles and soluble factors that together provide a broad array of bioactive molecules, including cytokines, growth factors, and microRNAs, associated with the regulation of numerous physiological processes. Indeed, the importance of the MSC secretome on liver regeneration has been recognized in several preclinical models of hepatic diseases [18,84,85].

In this work, we evaluated the therapeutic capacity of the secretome derived from human MSCs to attenuate DILI induced by exposure to AMI or TMX.

DILI is usually classified under two categories referred to as “intrinsic” and “idiosyncratic”. Although there is no universal definition of either, the first one is dose-dependent and predictable, since the toxicity is attributed to the chemical properties of the drug rather than to some particular aspect of the biology of the affected individual. Acetaminophen (APAP), hydrogen peroxide (H_2_O_2_), and carbon tetrachloride (CCl_4_) belong to this category [86,87,88]. On the other hand, idiosyncratic DILI is described as not dose dependent and unpredictable, and its pathogenesis is not fully understood; both AMI and TMX are included in this group [89].

Previous studies have evaluated the hepatoprotective effect of MSCs or the MSC secretome in models of DILI induced by exposure to APAP [19,85,90,91,92], H_2_O_2_ [85,92], or CCl_4_ [93,94]. However, to the best of our knowledge, this work represents the first study using the secretome obtained from human MSCs for the treatment of idiosyncratic DILI induced by AMI or TMX. In the same line, the hepatoprotective effects of MSC secretome administration in a combined model of NAFLD and DILI have not been previously reported. In concordance with previous reports in which the MSCs or MSC secretome was evaluated after APAP or CCL_4_ exposure (intrinsic DILI models), we described both an increase in cell viability and a decrease in ROS production. Additionally, in the present study, the MSC secretome administration prevented the NASH transition in an animal model of hepatic steatosis plus the chronic administration of AMI. This was probably related to an improvement in the lipid metabolism of hepatocytes [13,58].

For the design of an accurate model of DILI, it is important to consider that cytotoxicity is directly related to the capacity of the cells to metabolize xenobiotics. As mentioned above, human hepatocytes are disadvantageous because liver samples are scarce, and primary cultures show in vitro instability and high variability in their content of CYP450 enzymes among donors [31,95]. These difficulties have led to the use of hepatic cell lines as in vitro models of DILI and the exploration of therapeutic agents being widely reported in the literature.

The use of HepG2 cells in toxicological studies is controversial. On the one hand, several studies have reported their poor CYP450 basal level and reduced activity of numerous xenobiotic metabolizing enzymes, which could provide misleading results in toxicity evaluations of compounds that require biotransformation [35,36]. On the other hand, multiple studies have shown that the inducibility of CYP 1A2 and 3A4 gene expression in HepG2 cells is similar to that of human hepatocytes, and they have been used successfully in toxicity tests [33,37,40,42]. Schoonen et al. classified 70% of the compounds with known toxicity as cytotoxic using HepG2 cells [96,97], whereas O’Brien et al. detected cytotoxicity with 80% sensitivity and 90% specificity in HepG2 cells [98]. Both groups included AMI and TMX in their studies.

Alternatively, HepaRG cells have been proposed as a surrogate of primary hepatocytes for xenobiotic metabolism and toxicity studies. Moreover, when these cells are cultured in the presence of DMSO, they present high levels of CYP450 1A2, 2B6, and 3A4 [42,43,99,100]. In the present work, we used both cell models, in a complementary way, to strengthen the evidence of the therapeutic effect of the MSC secretome against DILI induced by TMX or AMI [101]. In our laboratory, HepaRG cells were more susceptible to AMI than HepG2 cells. In the case of TMX, the difference between both cell types was less evident; however, the toxicity induced by TMX tended to be higher in HepaRG cells than in HepG2 cells.

These results are in accordance with the increased gene expression in HepaRG cells of the most relevant CYP450 enzymes involved in the biotransformation of AMI and TMX. While CYP 1A1, 2D4, 2B6, and particularly 34A, are responsible for the conversion of AMI into MDEA and DDEA, its two main metabolites [56,63,102], CYP 1A1, 1A2, 2D6, 2C9, and 3A4, contribute to the production of the toxic metabolites of TMX, 4-hydroxy-tamoxifen and N-desmethyl-tamoxifen [103,104,105]. Although it is known that the mRNA level does not necessarily correlate well with the enzymatic activities of the encoded protein due to posttranscriptional regulation, in the case of these CYP450 enzymes, this correlation has been previously demonstrated [66].

Another factor that could be related to the increased resistance of HepG2 cells, at least partially, is their marked capacity to switch between oxidative and glycolytic metabolism depending on the cellular environment such as the presence of mitochondrial toxicants such as AMI or TMX [106,107].

In the present study, both drugs induced a significant reduction in cell viability in a concentration-dependent manner, whereas coincubation with MSC secretome prevented this response under all conditions tested. In the same line, the release of cytochrome C into the cytoplasm with caspase 3/7 activation, as well as the release of LDH, indicate that both apoptosis and necrosis were evident in both cell types after exposure to AMI or TMX [69]. However, coincubation with MSC secretome prevented these responses at different degrees, with a concomitant decrease in the intracellular ROS production.

Interestingly, in the present work, exposure to AMI or TMX was associated with a clear and more significant reduction in cell viability (accompanied by an evident hepatoprotective effect) when compared to caspase activity or LDH release. Although the use of MTT as a cell viability marker has been widely validated in the in vitro models used in this work, it is important to keep in mind that this protocol is based on the metabolism of mitochondria [34,55]. In that sense, mitochondrial metabolism could be more sensitive than cytotoxicity for assessing the cellular effects of toxicants and the MSC secretome [40,43].

Cell death is an extreme condition, and it may not be the only end point to detect hepatotoxicity and to evaluate the effects of the MSC secretome. Thus, we evaluated the potential of MSC secretome to induce hepatocyte proliferation and the expression of factors that triggers growth-arrested injured cells to reenter the cell cycle.

Both IL-6 and TNF-α are key factors in initiating liver regeneration by committing cells to cell cycling, while iNOS mediates the formation of nitric oxide, which catalyzes the rate limiting step in the synthesis of prostaglandins that control regenerative processes [71,72,73]. When the hepatic regenerative process is induced, the resident immune cells, such as the Kupffer cells, are the primarily producer of IL-6 and TNF-α used for stimulating acute phase of proliferation [2]. However, in addition to immune cells, hepatocytes may also serve as a direct source of IL6 and TNF-α, i.e., the regenerative hepatic response also has an autocrine component [108].

In our in vitro models, the MSC secretome induced an increase in the proliferation of HepaRG cells, in agreement with the gene expression of IL-6, TNF-α, and iNOS.

It is important to note, the controversial role of TNF-α in hepatic regeneration. On the one hand, TNF-α mediates hepatocyte apoptosis and liver failure in diverse toxicity models [109]. On the other hand, as mentioned above, the loss of TNF-α function delays hepatocyte proliferation [110]. It is likely that TNF-α signaling on cells already “primed” to survive by the presence of growth factors can promote the same pathways [111]. This condition is presented when the MSC secretome is present, whereas in the absence of such signals, TNF-α then promotes death pathways.

Interestingly, although previous results showed a cytoprotective effect on HepG2 cells, exposure to the MSC secretome did not induce a significant increase in cell proliferation. This result can be explained by the proliferative nature of HepG2 cells which results in high energy expenditure when compared with differentiated HepaRG cells, limiting the proliferative potential under the stress conditions induced by AMI and TMX [112]. Alternatively, HepG2 cells are able to switch to glycolysis when oxidative phosphorylation (OXPHOS) is reduced (Crabtree effect), for example due to mitochondrial damage induced by AMI or TMX, in order to produce ATP to continue survival [107,113]. Although glycolysis is less efficient in terms of ATP production than OXPHOS, it is sufficient for cell survival, although not for cell proliferation [114].

At therapeutic dosages, AMI reaches plasma concentrations in the range of ≈2 µM, while reaching concentrations that are 10–20 times higher in the liver [115,116]. On the other hand, concentrations of up to 20 µM are achieved in the liver tissue of patients during steady-state tamoxifen treatment [117], suggesting that the results of this study are clinically relevant.

As proof of concept, we evaluated the hepatoprotective effect of the MSC secretome in a mouse model chronically exposed to AMI. To mimic the human administration protocol of AMI in the treatment of atrial fibrillation, the dose administered to the animals was in accordance with previous work, and was calculated according to the formula established by Reagan-Shaw et al. [118,119,120,121].

In a first approach, we administered AMI to normal mice. However, the experimental protocol used did not induce evident hepatic alterations. This result was not unexpected, since mitochondrial toxicity is difficult to mimic in vivo because mitochondria in regular rodent models are healthy and have a large respiratory capacity [122]. Our observations agree with studies carried out by Kikkawa et al. [123] and Takai et al. [124], who used higher AMI concentrations (300 mg/kg and 1000 mg/kg) for shorter periods (24–48 h), without evident liver injury.

It has been widely reported that some pathological conditions can overwhelm or inhibit the intrinsic regenerative potential of the liver [2]. In particular, patients with obesity and/or hepatic steatosis have a markedly higher risk of developing DILI [8,9,10,11]. With this in mind, we developed a mouse model of obesity generated by using a long-term exposition to HFD, which was subsequently exposed chronically to AMI. The HFD mouse model recapitulates many features of human NAFLD, including obesity and hepatic steatosis, but also insulin resistance, dyslipidemia, and, remarkably, impaired liver regeneration [6,7,48,58].

Histological examination of liver sections revealed a regular liver architecture (aside from steatosis) in untreated obese mice, whereas obese mice treated with AMI presented a severe disruption of liver architecture with macrosteatosis, foci of inflammatory cells, and ballooning degeneration of hepatocytes. Remarkably, cellular ballooning in NAFLD, defined as cellular enlargement 1.5–2 times the normal hepatocyte diameter, with rarefied cytoplasm, is one of the principle histological findings used to identify the presence of significant and potentially progressive NASH [125]. Interestingly, administration of MSC secretome prevented this transition. This hepatoprotective effect was not related to a reversion of the metabolic condition, since mice remained obese and hypercholesterolemic, with hepatic steatosis.

NAFLD represents a spectrum of disorders ranging from simple steatosis to steatosis with hepatocyte damage, leukocyte infiltration, and fibrosis, referred as nonalcoholic steatohepatitis (NASH), which may progress to cirrhosis. To explain the progression of the disease, the most accepted theory is the two-hits hypothesis [126]. In our experimental model, the fatty liver is merely the first hit, which renders the liver more susceptible to a second hit [127], given by the administration of AMI and its hepatotoxic effects [60,128]. Complementing the picture, the HFD increases the CYP450 levels in response to elevated liver concentrations of ketones and FFAs that serve as inducers and substrates of these enzymes [129,130]. In that sense, the heightened vulnerability of the hepatic tissue could be related, at least in part, to the increased production of the AMI metabolites DDEA and MDEA.

Qualitatively, fat deposition in the liver can be classified as micro or macrosteatosis. In our study, obese mice that received MSC secretome did not shift to predominant macrosteatosis as observed in obese animals treated with AMI. Although the nature of the shift from micro to macrosteatosis is unknown, it has been suggested that it relates to an alteration of fatty acid catabolism [131], and it has been associated with the progression of NAFLD to an impairment of hepatic functions [132].

AMI and its metabolites have been shown to impair β-oxidation by inhibiting carnitine palmitoyltransferase (CPT-1). As a consequence, FFAs and triglycerides accumulate in the cytoplasm and may be toxic in hepatocytes. Complementing these effects, AMI uncouples oxidative phosphorylation and increases the production of ROS, eventually leading to apoptosis [22,133,134].

Additionally, previous reports have shown that MSC administration can ameliorate NAFLD and protect hepatocytes from lipotoxicity. Li et al. demonstrated in db/db mice the upregulation of genes related to fatty acid oxidation (such as Acox-1, Ppar-α, and Cpt-1) and the downregulation of lipogenesis-related genes (such as Acc1 and Fas), after MSC administration [135]. We reported similar results in the mouse model of NAFLD induced by a long-term HFD exposition [58], while Chen et al. showed that MSC therapy improved lipid metabolism in HFD rats by improving intracellular calcium homeostasis and reducing endoplasmic reticulum (ER) stress [136].

Other important factors involved in NAFLD progression include increased production of proinflammatory and fibrogenic cytokines by parenchymal and nonparenchymal hepatic cells [126]. The factors that trigger the recruitment and activation of cells of the immune system have not been fully identified; however, damaged hepatocytes release DAMPs, which induce a proinflammatory activation of innate immune cells, thereby contributing to the pathogenesis of DILI [81,137]. In line with these findings, Takai et al. demonstrated in a mouse model that macrophages are involved in the enhancement of oxidative stress and liver injury induced by AMI by treating mice with GdCl3 before the administration of AMI, which significantly prevented the increase in hepatic liver injury [124]. Alternatively, neutrophils, Kupffer cells and HSCs have CYP450 enzymes capable of generating reactive metabolites that, in turn, activate innate immune responses themselves [138,139].

In this study, obese animals that received AMI presented a significant increase in the number of macrophages per tissue field. However, the administration of MSC secretome highly diminished the cellular infiltrate, which was similar to that seen in obese control animals. This result is not unexpected, since the hallmark of MSCs is their anti-inflammatory and immunomodulatory activity both in vitro and in vivo [140].

Fat deposition, repeated chronic injury, and the proinflammatory microenvironment lead to the activation of HSCs and differentiation into collagen-secreting myofibroblasts, extracellular matrix deposition, and tissue fibrosis [126]. In our experimental model, although no obvious fibrosis was identified in any experimental group, obese animals treated with AMI presented an increased density of myofibroblasts in the tissue (α-SMA positive cells), which represents the initial stage in the process of fibrogenesis.

The use of MSC secretome to reduce liver fibrosis has gradually gained importance in regenerative medicine since it has been related to both a reduction in proliferation and an induction of apoptosis in HSCs [141,142,143].

In the same line, fibrosis has been associated with the epithelial–mesenchymal transition (EMT) process, driven by the TGF-β/Smad-signaling pathway [144,145]. MSCs also inhibit TGF-β/Smad signaling and affect EMT-associated proteins in the liver, causing a decrease in α-SMA and vimentin in HSCs [146,147]. These processes are modulated by factors and cytokines within the MSC secretome, such as TGF-β3, HGF, MFGE8, IL-10, and TNF-α [143,146,148]. Therefore, inhibition or reversal of EMT may be one of the mechanisms by which the MSC secretome prevented hepatic fibrosis in the present study.

As previously described, exposure to AMI induced hepatocyte apoptosis [124]. However, liver sections from animals treated with AMI plus MSC secretome showed a significant reduction in cell death and increased proliferation of hepatocytes was seen in this group of animals. This effect is in line with the in vitro findings in which the MSC secretome was able to sustain higher cell viability after exposure to both AMI and TMX, and with previous reports that describe the improvement of the hepatic proliferative response in the fatty liver after MSC administration [13].

In the present study, the serum levels of ALT and AST did not correlate closely with the histologic features of the respective groups. In that sense, it is the damage of the hepatocyte membrane, more than hepatocyte necrosis or apoptosis, which induces the release of transaminases into the bloodstream. In our model, as in similar studies, the degree of hepatocyte death and the level of aminotransferases were found to correlate poorly [123,149,150].

In recent years, it has become evident that the hepatoprotective effect of MSCs is mediated by the paracrine secretion of factors which have not been fully characterized [151].

To identify the candidate mechanisms by which the factors present in the MSC secretome could alleviate TMX or AMI induced DILI, we performed a proteome analysis by mass spectrometry. For a better understanding, the proteins were distributed over a wide array of biochemical and cellular processes related to the effects described in the present study, such as (i) factors related to the cell cycle control and proliferation; (ii) growth factors and organization of the extracellular matrix; (iii) cytokines and immune response, and (iv) cell metabolism and lipid metabolism. However, in accordance with previous reports, we identified a row of MSC derived factors with pleiotropic actions that might protect or potentiate liver regeneration via multiple and interconnected pathways. For example, among the factors secreted by MSCs, both vascular endothelial growth factor (VEGF) and angiopoietins (ANG), which promote vascularization and mitogenesis in the injured liver [2,151], were identified.

The IL-6 present in the MSC secretome induces hepatocyte proliferation, and enhances gene expression of chitinase 3-like protein 1 in HSCs, which has been associated positively with cell survival and negatively with liver fibrosis [148,152].

Various factors associated with the recovery of the capacity of hepatocytes to proliferate were identified. For example, IGFBPs and Serpin E1 secreted by MSCs seem to contribute to tissue remodeling and morphogenesis, thereby promoting liver regeneration under impaired conditions [151,153]. Milk factor globule EGF 8 protein (MFGE8) reduces hepatocyte apoptosis by inhibiting the activation of the IRE1α/ASK1/JNK pathway and promotes hepatocyte proliferation by phosphorylation of ERK and AKT [154]. At the same time, MFGE8 is a strong inhibitor of HSCs: it prevents liver fibrosis by downregulating the TGF-β type 1 receptor [155].

The hepatoprotective properties of platelet-derived growth factor (PDGF) have been described in preclinical models and patients [156,157]. While pentraxin 3 (PTX3) plays an essential role in the regulation of innate immunity, inflammation, and matrix deposition [158], its crosstalk with signaling growth factors such as HGF and EGF convert it into a key player in liver morphogenesis and regeneration after liver injury [159].

MSCs participate in remodeling the extracellular matrix (ECM). The matrix metalloproteases (MMPs), found in abundance in the MSC secretome, are involved in the breakdown of the extracellular matrix and prevent the differentiation of fibroblasts to myofibroblasts [160]. Previous studies have demonstrated that C1q/tumor necrosis factor-related protein-3 (CTRP3) prevents the activation of HSCs both in vitro and in vivo, inhibiting the Notch-1/jagged-1 signaling pathway [161].

As described above, the MSC secretome includes a diverse range of soluble factors, but also extracellular microvesicles (MVs). MVs are enriched in bioactive molecules such as proteins, lipids, mRNA, and miRNA [162]. In that sense, several miRNAs derived from MSC-MVs have been related to their hepatoprotective effect [163]. For example, miR-223 reduced inflammation in a mouse model of autoimmune hepatitis [164], whereas, miR-122 and miR-181 alleviated liver fibrosis and collagen deposition in a mouse model of CCl4-induced liver fibrosis [165,166]. Finally, the results described in the present work may result from either type of constituents—soluble factors and MVs—or both.

Even if possible, dissecting the MSC secretome to identify a single molecular pathway to serve as therapeutic target does not seem to be a reasonable strategy to address the treatment of complex liver diseases. Unlike most pharmacological treatments, the MSC secretome can act on multiple pathways, enhancing its therapeutic effect [18]. However, the understanding of these interactions would allow the enrichment of therapeutic factors and thereby maximize the therapeutic effects for a specific liver pathophysiology [75].

## 5. Conclusions

In conclusion, the present study provides proof of the concept that treatment with the MSC secretome confers in vitro and in vivo hepatoprotection after drug-induced liver injury by AMI or TMX. This effect is probably mediated by the maintenance of tissue homeostasis and the recovery of the hepatocyte proliferation capacity, particularly when this response is pathologically impaired.

Taking into account the limited availability of conventional liver transplants, this treatment may represent a novel adjunctive therapy for DILI induced by TMX or AMI. Thus, the present work supports conducting further studies with the MSC secretome oriented to clarify its mechanisms of action, optimize its isolation and therapeutic efficacy, and define the appropriate protocols for its therapeutic applications to prevent liver failure associated with TMX or AMI injury.

## Data Availability

Not applicable.

## Supplementary Materials

The following supporting information can be downloaded at: https://www.mdpi.com/article/10.3390/cells12040636/s1, Table S1: RT-qPCR specific primers and characteristics of amplicons. Table S2: List of complete secretome proteins identified. Figure S1: Effect of MSC secretome on liver cells viability. Figure S2: Cytotoxicity of amiodarone (AMI) or tamoxifen (TMX) in HepG2 and HepaRG cells. Figure S3: Cytochrome P450 mRNA levels in HepG2 and HepaRG cells. Figure S4: Coincubation with MSC secretome prevented cytochrome C release in HepG2 cells exposed to AMI or TMX. Figure S5: Quantitative analysis of cytochrome C release by flow cytometry in HepG2 and HepaRG cells exposed to AMI or TMX alone or coincubated with MSC secretome. Figure S6: MSC secretome presented a cytoprotective effect in HepaRG cells incubated with TMX. Figure S7: Coincubation with MSC secretome not change HepG2 proliferation rate after exposure to AMI or TMX. Figure S8: Histological and biochemical characterization of normal mice treated with AMI. Figure S9: Evaluation of liver fibrosis and biochemical profile in obese mice treated with AMI alone or AMI plus MSC secretome. Figure S10: MSC secretome administration prevented hepatic histopathological alterations induced by AMI in an obese mouse model [59,125]. Figure S11: Colocalization of hepatocyte and proliferation or apoptotic markers. Figure S12: MSC secretome administration inhibited apoptosis if hepatic cells after chronic exposure to AMI.
