# Supplementary material for: Administration of Secretome Derived from Human Mesenchymal Stem Cells Induces Hepatoprotective Effects in Models of Idiosyncratic Drug-Induced Liver Injury Caused by Amiodarone or Tamoxifen
Source: Cells. 2023 Feb 16;12(4):636. doi: 10.3390/cells12040636 (PMC9954258; doi:10.3390/cells12040636)
Supplement: Supplementary file 1 [file cells-12-00636-s001.zip › Figure S1.pdf]

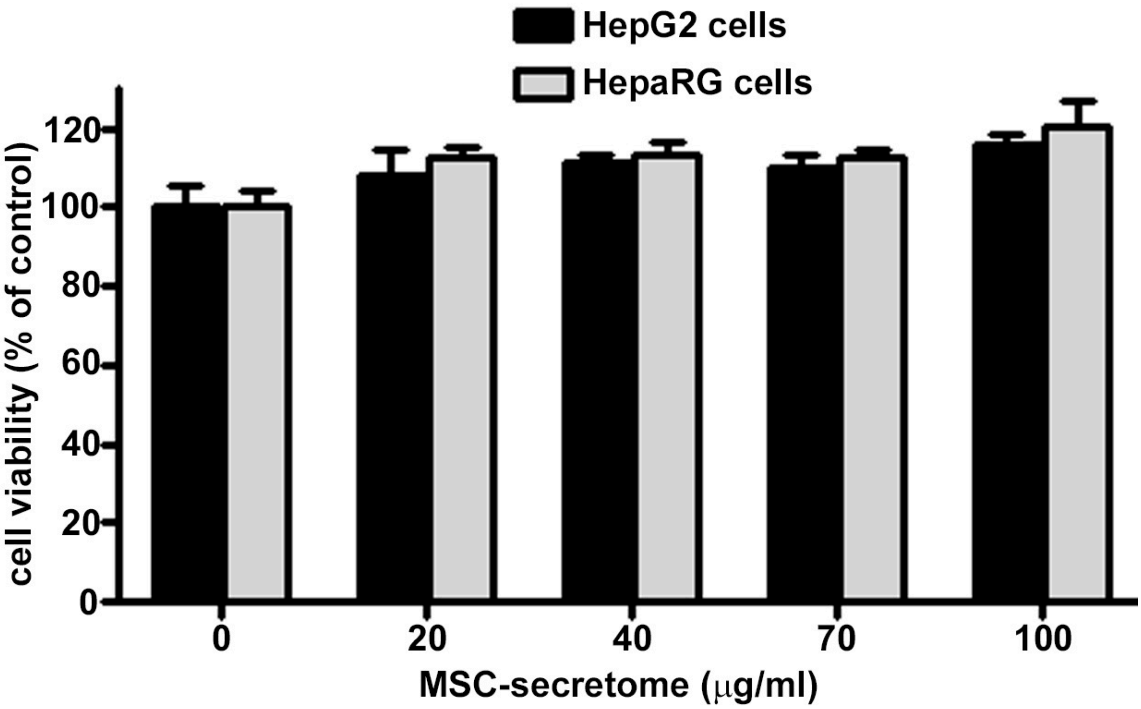

Figure S1: Effect of MSC secretome on liver cells viability. HepG2 and HepaRG cells were incubated with different concentrations of MSC secretome (from 20 to 100 µg/ml) and a viability MTT assay was performed 24 hours later. Cell viability was normalized against the secretome vehicle (PBS) control group and expressed as percentage. Data are presented as means ± SEM (n=3) of three independent experiments.
