# Supplementary material for: Administration of Secretome Derived from Human Mesenchymal Stem Cells Induces Hepatoprotective Effects in Models of Idiosyncratic Drug-Induced Liver Injury Caused by Amiodarone or Tamoxifen
Source: Cells. 2023 Feb 16;12(4):636. doi: 10.3390/cells12040636 (PMC9954258; doi:10.3390/cells12040636)
Supplement: Supplementary file 1 [file cells-12-00636-s001.zip › Figure S10.pdf]

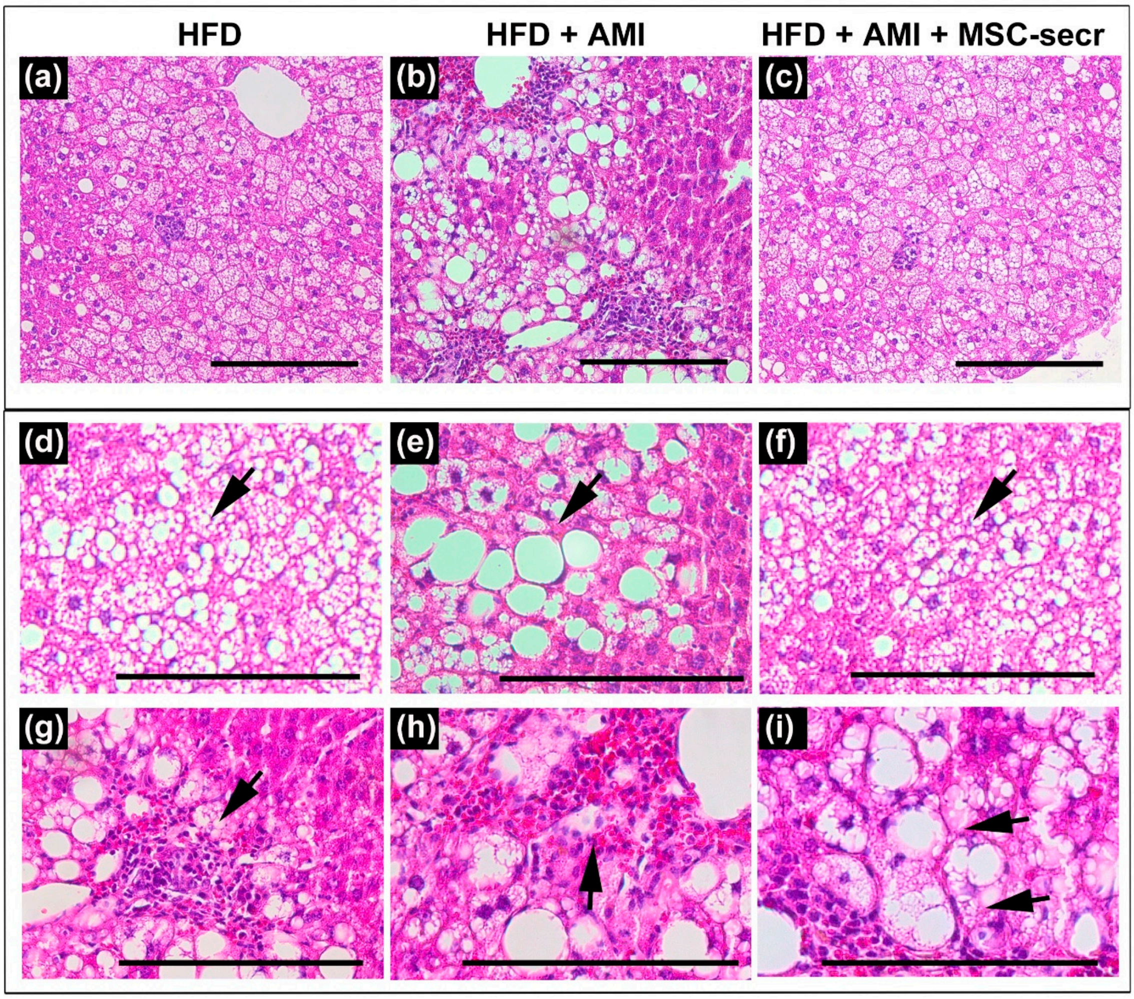

(j)

Treatment	HFD	HFD + AMI	HFD + AMI MSC-secr
Steatosis	2.7 (0.2)	3 (0.0)	2.9 (0.1)
Inflammatory foci	0.6 (0.2)	2.5 (0.3) *	0.7 (0.1) #
Balloning	0.2 (0.1)	1.4 (0.3) *	0.3 (0.2) #
**NAFLD activity score**	**3.5 (0.3)**	**6.9 (0.2) ***	**3.9 (0.2) #**

Figure S10: MSC secretome administration prevented hepatic histopathological alterations induced by AMI in an obese mouse model. Male mice were fed a high fat diet (HFD) for 34 weeks and divided into three groups. During the last four weeks, one group did not receive additional treatment (HFD group), while a second group was treated daily with AMI (40 mg/kg) (HFD+AMI). The third group received MSC secretome endovenously once a week (HFD+AMI+secr) (Figure 7). Hematoxilin and eosin staining: representative micrographs of liver sections of **(a)** HFD; **(b)** HFD+AMI and **(c)** HFD+AMI+secr groups.

Qualitatively, fat deposition in the liver can be classified as micro or macrosteatosis. Microsteatosis consists of many small fatty inclusions in the cytoplasm, without

displacement of organelles. In contrast, in macrosteatosis hepatocytes contain one single vacuole of fat that displaces the nucleus to the periphery of the cell. **(d)** HFD group presented predominant microsteatosis, and **(f)** obese mice that received MSC secretome did not shift to predominant macrosteatosis as observed **(e)** in HFD+AMI group. Additionally, the HFD+AMI group presented **(g)** multiple inflammatory foci, **(h)** necrosis around central vein and **(i)** hepatocellular ballooning. Interestingly, cellular ballooning in NAFLD, defined as cellular enlargement 1.5-2 times the normal hepatocyte diameter, whit rarefied cytoplasm, is one of the principle histological findings used to identify the presence of significant and potentially progressive NASH [1]. Arrows indicate the mentioned histopathological changes. Bars represent 200 μm.

(j) Score to evaluate NAFLD activity [2]. *p<0.05 *vs.* control HFD mice; #p<0.05 *vs.* HFD+AMI mice. 30 random fields per animal and six animals per group were analyzed.

Reference List

[1] Caldwell S, Ikura Y, Dias D, et al. Hepatocellular ballooning in NASH. J Hepatol 2010;53:719-23.

[2] Kleiner DE, Brunt EM, Van NM, et al. Design and validation of a histological scoring system for nonalcoholic fatty liver disease. Hepatology 2005;41:1313-21.

[3] El-Badry AM, Graf R, Clavien PA. Omega 3 - Omega 6: What is right for the liver? J Hepatol 2007;47:718-25.

[4] Selzner N, Selzner M, Jochum W, Amann-Vesti B, Graf R, Clavien PA. Mouse livers with macrosteatosis are more susceptible to normothermic ischemic injury than those with microsteatosis. J Hepatol 2006;44:694-701.
