# Supplementary material for: Administration of Secretome Derived from Human Mesenchymal Stem Cells Induces Hepatoprotective Effects in Models of Idiosyncratic Drug-Induced Liver Injury Caused by Amiodarone or Tamoxifen
Source: Cells. 2023 Feb 16;12(4):636. doi: 10.3390/cells12040636 (PMC9954258; doi:10.3390/cells12040636)
Supplement: Supplementary file 1 [file cells-12-00636-s001.zip › Figure S11.pdf]

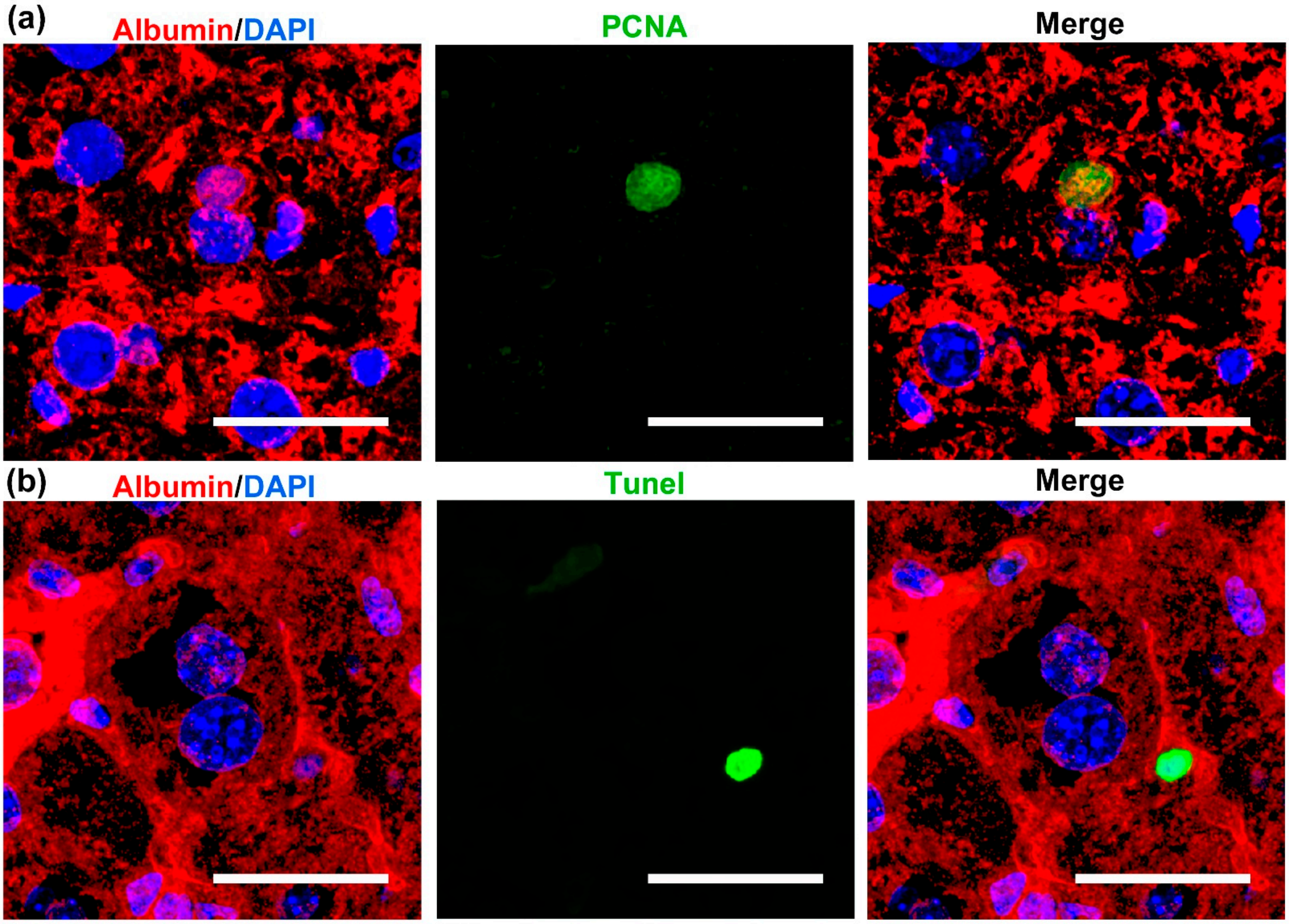

Figure S11: Colocalization of hepatocyte and proliferation or apoptotic markers.
Hepatocyte proliferation was identified by colocalization of PCNA (Alexa Fluor 488, green) and albumin (Alexa Fluor 555, red), while hepatocyte apoptosis was evaluated by colocalization of TUNEL (FITC, green) and albumin. Nuclei were counterstained with DAPI (blue). Representative confocal microscopy micrographs of **(a)** proliferation and **(b)** apoptosis, 24 hours after the last administration of MSC secretome. Bars represent 20 µm.
