# Supplementary material for: Administration of Secretome Derived from Human Mesenchymal Stem Cells Induces Hepatoprotective Effects in Models of Idiosyncratic Drug-Induced Liver Injury Caused by Amiodarone or Tamoxifen
Source: Cells. 2023 Feb 16;12(4):636. doi: 10.3390/cells12040636 (PMC9954258; doi:10.3390/cells12040636)
Supplement: Supplementary file 1 [file cells-12-00636-s001.zip › Figure S12.pdf]

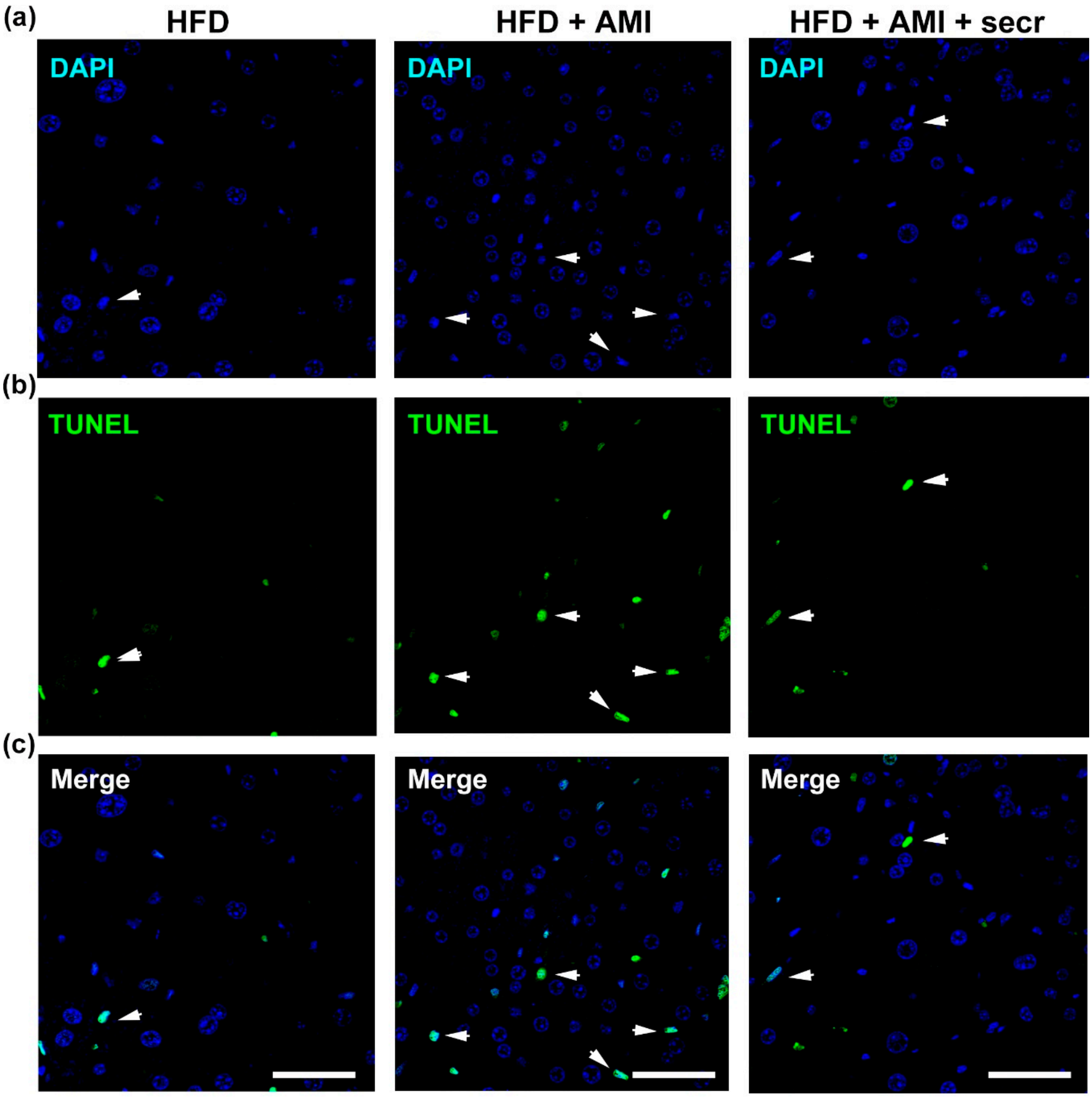

Figure 12. MSC secretome administration inhibited apoptosis if hepatic cells after chronic exposure to AMI. Male mice were fed a high fat diet (HFD) for 34 weeks and divided into three groups. During the last four weeks, one group did not receive additional treatment (HFD group), while a second group was treated daily with AMI (40 mg/kg) (HFD+AMI). The third group received MSC secretome endovenously once a week (HFD+AMI+secr). Cell apoptosis was analyzed in all experimental groups 24 hours after the last administration of AMI (Figure 8). **(a)** The nuclei were stained with DAPI (blue), **(b)** while the effect of MSC secretome on cell apoptosis was determined by TUNEL staining (FITC, green). **(c)** colocalization of both stains. Arrows indicate TUNEL (+) nuclei. Scale bars represent 50 μm.
