# Supplementary material for: Administration of Secretome Derived from Human Mesenchymal Stem Cells Induces Hepatoprotective Effects in Models of Idiosyncratic Drug-Induced Liver Injury Caused by Amiodarone or Tamoxifen
Source: Cells. 2023 Feb 16;12(4):636. doi: 10.3390/cells12040636 (PMC9954258; doi:10.3390/cells12040636)
Supplement: Supplementary file 1 [file cells-12-00636-s001.zip › Figure S2.pdf]

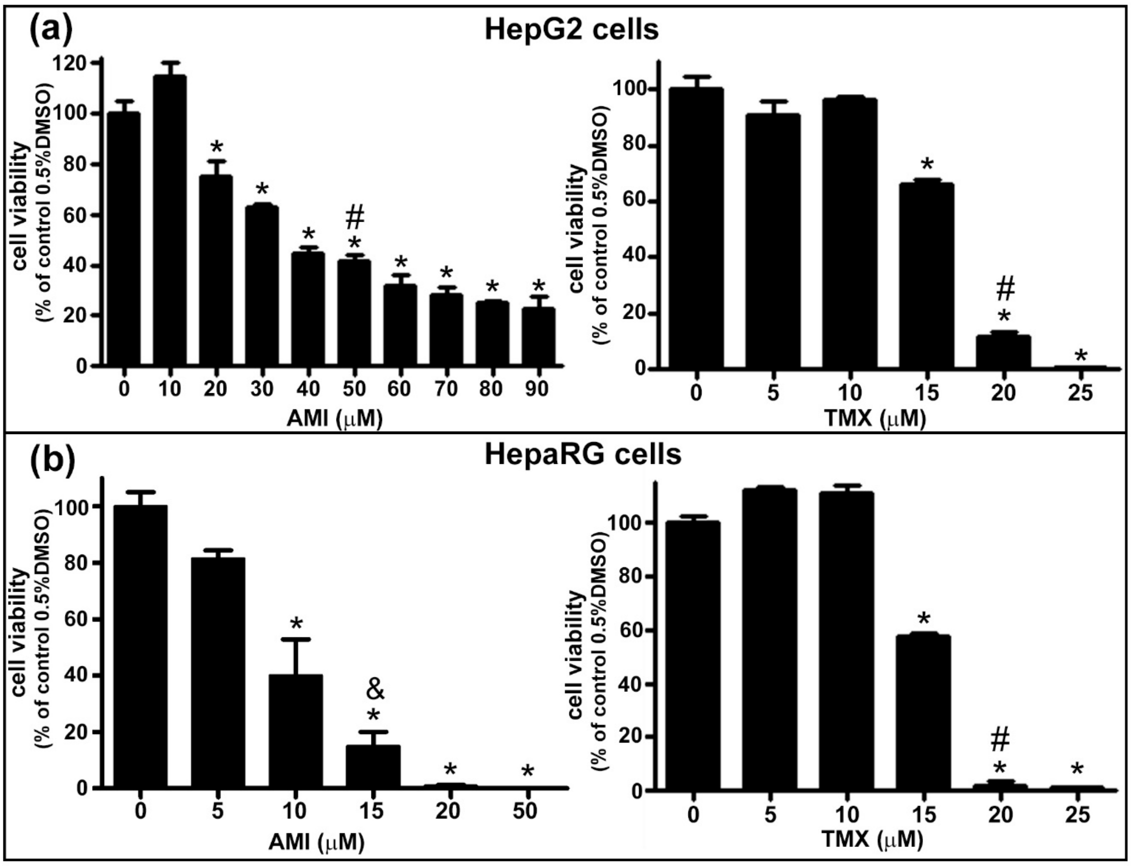

Figure S2: Cytotoxicity of amiodarone (AMI) or tamoxifen (TMX) in HepG2 and HepaRG cells.

The cytotoxicity of AMI and TMX in **(a)** HepG2 and **(b)** HepaRG cells was evaluated. Both cell lines were treated with AMI or TMX at the indicated concentrations for 24 hours. As a control group, cells were incubated with drug solvent alone (0.5% DMSO). Cell viability was measured by an MTT assay at the end of the treatment, and control group values were taken as 100%. Data are presented as means ± SEM (n=3) of three independent experiments. *: $p<0.05$ *vs.* control (0.5% DMSO); #: $p<0.05$ *vs.* TMX (15 μM); &: $p<0.05$ *vs.* AMI (10 μM).
