# Supplementary material for: Administration of Secretome Derived from Human Mesenchymal Stem Cells Induces Hepatoprotective Effects in Models of Idiosyncratic Drug-Induced Liver Injury Caused by Amiodarone or Tamoxifen
Source: Cells. 2023 Feb 16;12(4):636. doi: 10.3390/cells12040636 (PMC9954258; doi:10.3390/cells12040636)
Supplement: Supplementary file 1 [file cells-12-00636-s001.zip › Figure S3.pdf]

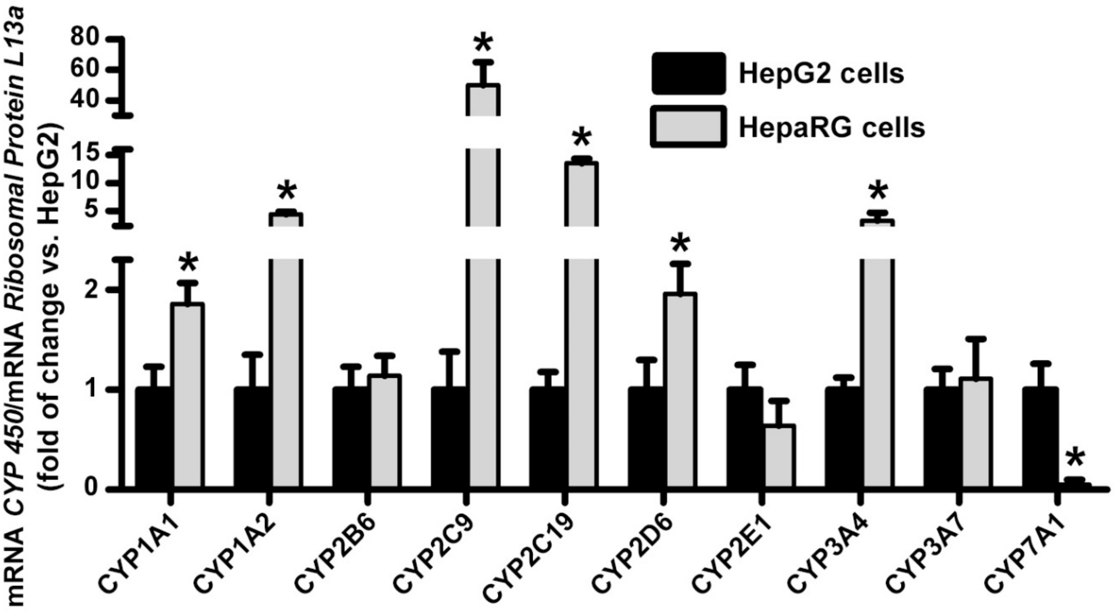

Figure S3: Cytochrome P450 mRNA levels in HepG2 and HepaRG cells.
CYP450 mRNA levels were measured by RT-qPCR in both HepG2 and HepaRG cell lines. The expression of CYP genes were normalized against that of the RPL13a gene and expressed as fold of change *vs.* that of HepG2 cells. All data are presented as means ± SEM (n=4). *: $p<0.05$ *vs.* HepG2 cells.
