# Supplementary material for: Administration of Secretome Derived from Human Mesenchymal Stem Cells Induces Hepatoprotective Effects in Models of Idiosyncratic Drug-Induced Liver Injury Caused by Amiodarone or Tamoxifen
Source: Cells. 2023 Feb 16;12(4):636. doi: 10.3390/cells12040636 (PMC9954258; doi:10.3390/cells12040636)
Supplement: Supplementary file 1 [file cells-12-00636-s001.zip › Figure S4.pdf]

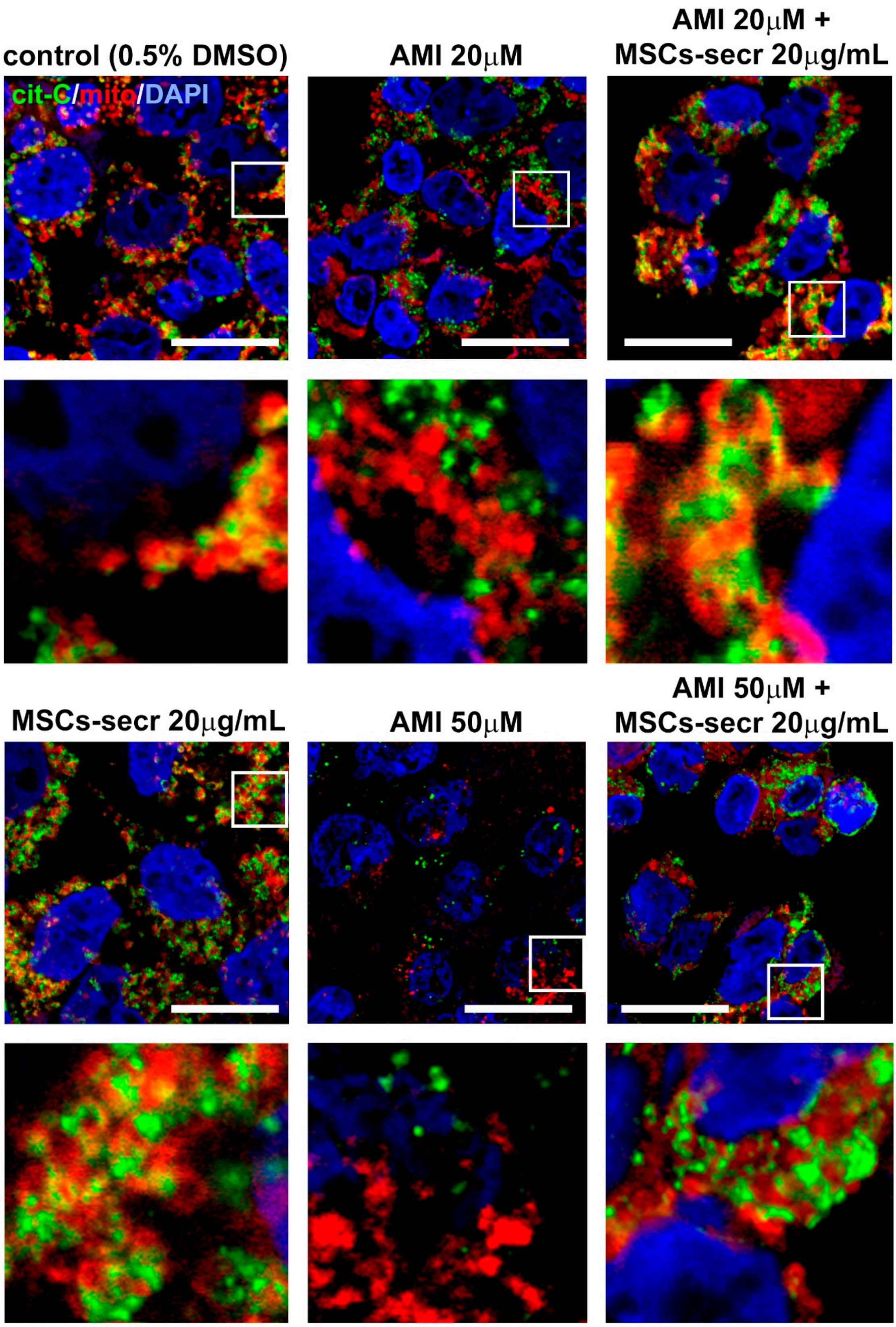
control (0.5% DMSO)
AMI 20μM
AMI 20μM +
MSCs-secr 20μg/mL
cit-C/mito/DAPI
MSCs-secr 20μg/mL
AMI 50μM
AMI 50μM +
MSCs-secr 20μg/mL

Figure S4: Coincubation with MSC secretome prevented cytochrome C release in HepG2 cells exposed to AMI or TMX. The mitochondrial release of cytochrome C was evaluated by immunofluorescense in HepG2 cells exposed to AMI or TMX alone or coincubated with MSC secretome (20µg/ml) for 24 hours (please see Figure 3). Representative micrographs of mitochondria (MitoTracker, red), cytochrome C (Cyt C, green), and their colocalization (merge, yellow); nuclei were counterstained with DAPI (blue). Cells with drug solvent (0.5% DMSO) was used as control group and showed maximal colocalization At the bottom of each image a magnification of each insert represented by the white rectangle is shown. Scale bars represent 20µm.
