# Supplementary material for: Administration of Secretome Derived from Human Mesenchymal Stem Cells Induces Hepatoprotective Effects in Models of Idiosyncratic Drug-Induced Liver Injury Caused by Amiodarone or Tamoxifen
Source: Cells. 2023 Feb 16;12(4):636. doi: 10.3390/cells12040636 (PMC9954258; doi:10.3390/cells12040636)
Supplement: Supplementary file 1 [file cells-12-00636-s001.zip › Figure S5.pdf]

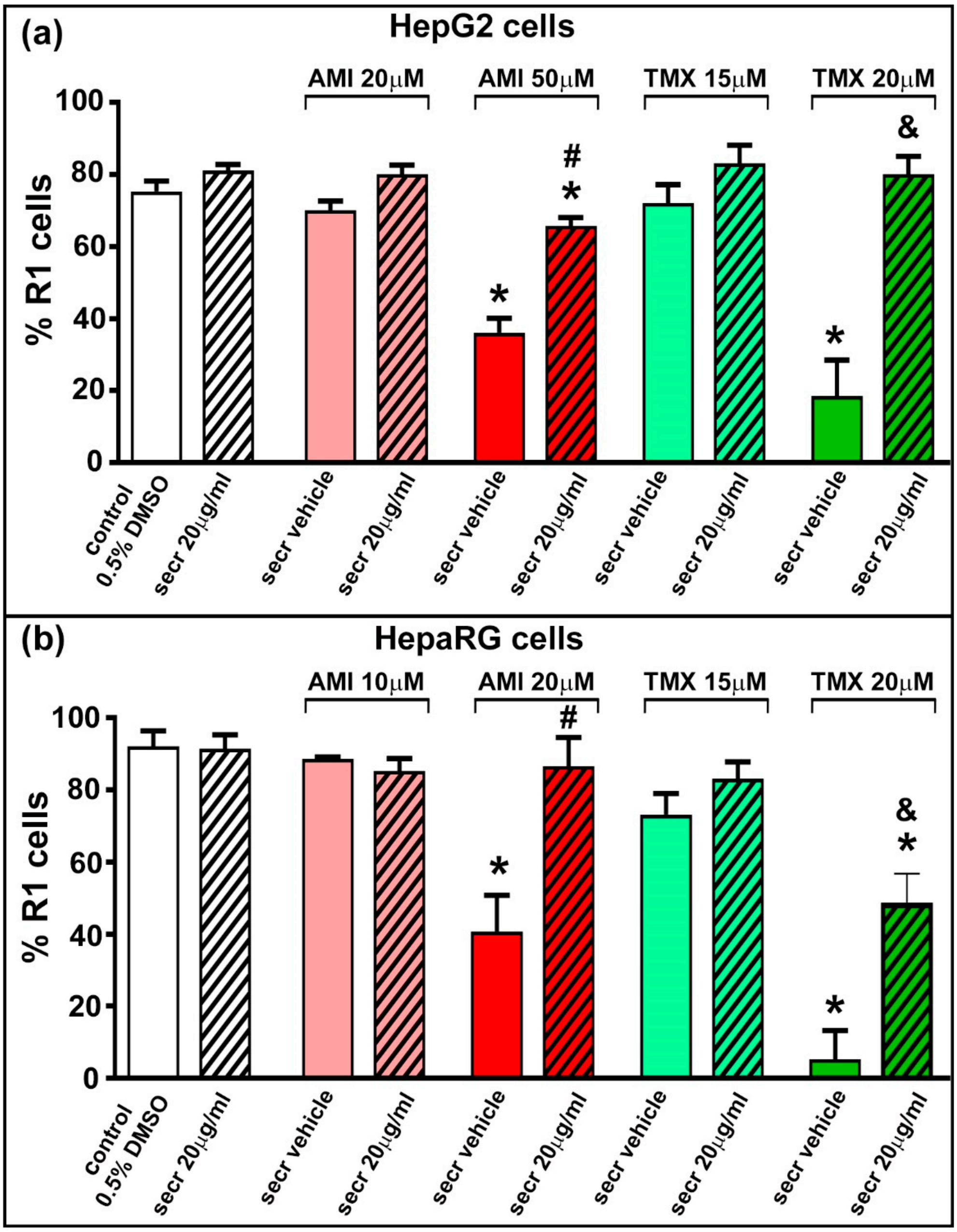


**Figure S5: Quantitative analysis of cytochrome C release by flow cytometry in HepG2 and HepaRG cells exposed to AMI or TMX alone or coincubated with MSC secretome.**

Cytochrome C release was evaluated quantitatively by flow cytometry. **(a)** HepG2 and **(b)** HepaRG cells were incubated with AMI or TMX alone or coincubated with MSC secretome for 24 hours. Cells were permeabilized with digitonin and labeled with anti-cytochrome C Alexa Fluor 488 antibody. Cells with drug solvent (0.5% DMSO) was used as control group and showed maximal cytochrome C fluorescence. The values of the percentage of cells in the R1 regions of the fluorescence histograms shown in Figure 3b

and 3d were plotted for each condition. Bars represent the mean ± SEM (n=4) of three independent experiments. *$p<0.05$ *vs.* control group (0.5% DMSO); #$p<0.05$ *vs.* AMI alone of the same experimental group (same AMI concentration); &$p<0.05$ *vs.* TMX alone of the same experimental group (same TMX concentration).
