# Supplementary material for: Administration of Secretome Derived from Human Mesenchymal Stem Cells Induces Hepatoprotective Effects in Models of Idiosyncratic Drug-Induced Liver Injury Caused by Amiodarone or Tamoxifen
Source: Cells. 2023 Feb 16;12(4):636. doi: 10.3390/cells12040636 (PMC9954258; doi:10.3390/cells12040636)
Supplement: Supplementary file 1 [file cells-12-00636-s001.zip › Figure S6.pdf]

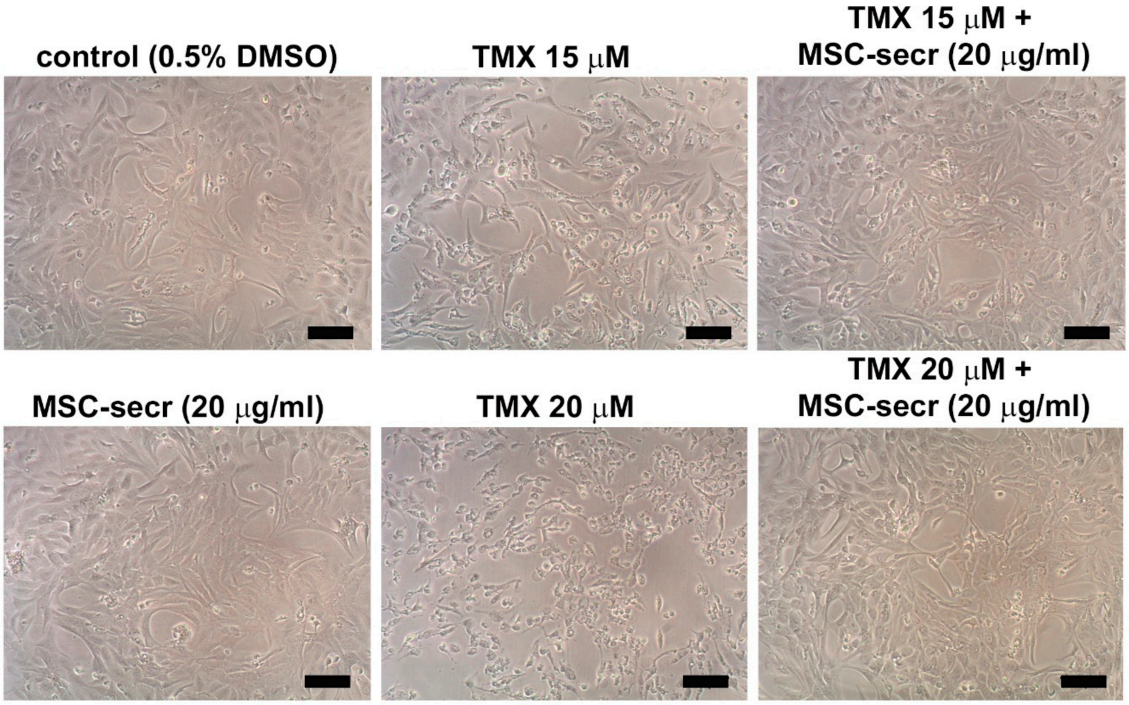

Figure S6: MSC secretome presented a cytoprotective effect in HepaRG cells incubated with TMX. Optical microscopic observation of the cytoprotective effect of MSC secretome on HepaRG cells exposed to TMX (15 and 20 µM), alone or coincubated with MSC secretome (20 µg/ml) for 24 hours. As control group, cells were incubated with the drug solvent (0.5% DMSO). Representative micrograph of three independent experiments. Bars represent 100µm.
