# Supplementary material for: Administration of Secretome Derived from Human Mesenchymal Stem Cells Induces Hepatoprotective Effects in Models of Idiosyncratic Drug-Induced Liver Injury Caused by Amiodarone or Tamoxifen
Source: Cells. 2023 Feb 16;12(4):636. doi: 10.3390/cells12040636 (PMC9954258; doi:10.3390/cells12040636)
Supplement: Supplementary file 1 [file cells-12-00636-s001.zip › Figure S7.pdf]

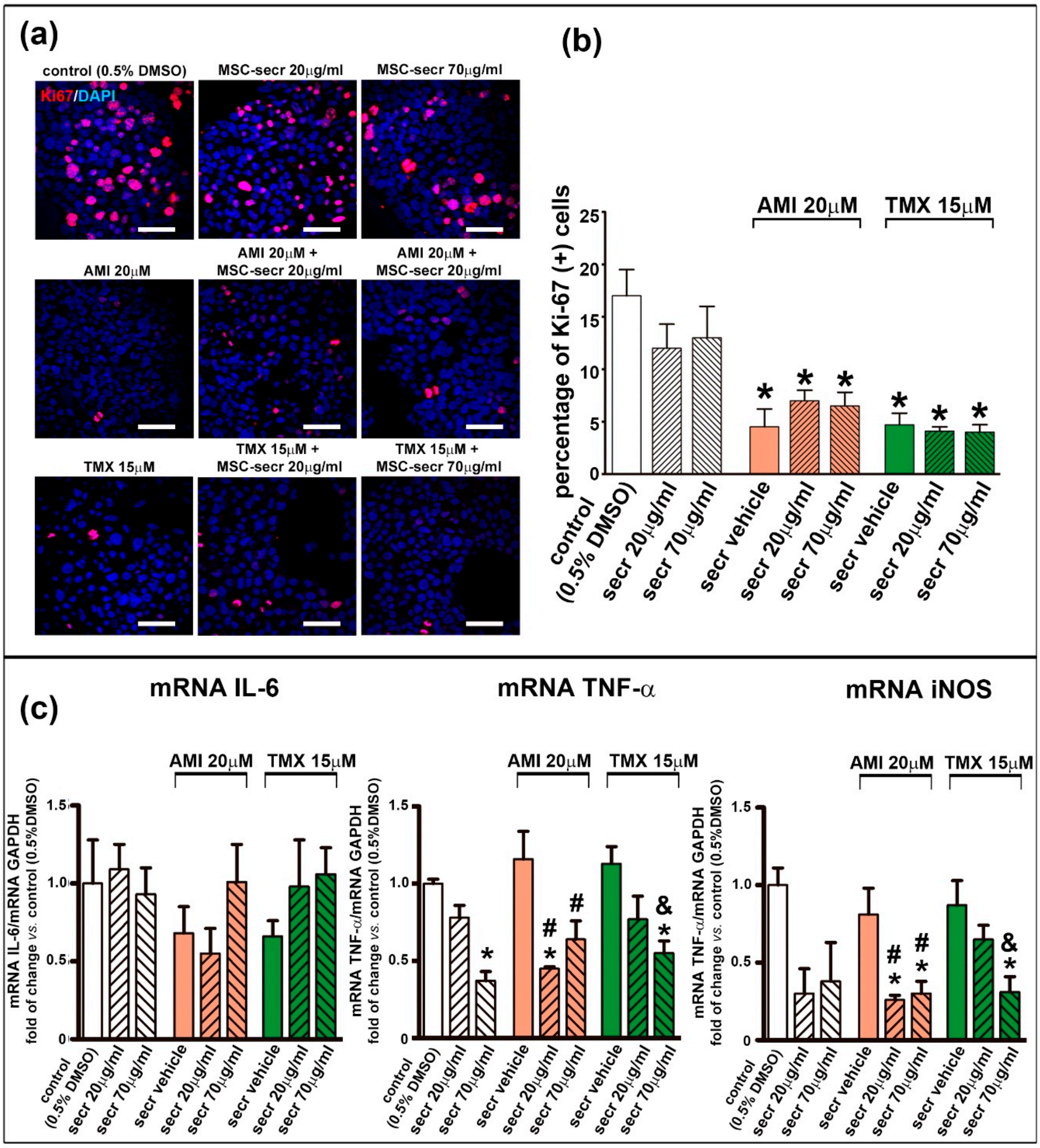

Figure S7: Coincubation with MSC secretome not change HepG2 proliferation rate after exposure to AMI or TMX. Cell proliferation was evaluated in HepG2 cells exposed to AMI or TMX alone or coincubated with two concentrations of MSC secretome (20 and 70 μg/ml) for 24 hours. As control group, cells were incubated with the drug solvent (0.5% DMSO). Ki-67 immunoreactivity (Alexa Fluor 555, red) was evaluated by immunofluorescence. Nuclei were counterstained with DAPI (blue). **(a)** Representative micrographs of HepG2 cells in each experimental condition. Scale bars represent 50μm. **(b)** Quantification of Ki-67 positive nuclei was done by digital image analysis. All data are presented as means ± SEM of Ki-67-positive nuclei per 100 hepatocytes in 20 high-power fields per slides and three replicates per experimental group. **(c)** Expression of

key factors of proliferative response after 24 hours of treatment, was evaluated by RT-qPCR, normalized against GAPDH and expressed as fold of change *vs.* control group (0.5% DMSO).

Data are presented as means ± SEM (n=4) of three independent experiments. *p<0.05 *vs.* control group (0.5% DMSO); #p<0.05 *vs.* AMI plus secretome vehicle, of the same experimental group (same AMI concentration); &p<0.05 *vs.* TMX plus secretome vehicle, of the same experimental group (same TMX concentration). **secr vehicle:** MSC secretome vehicle (PBS); **secr:** MSC secretome; **AMI**: amiodarone; **TMX**: tamoxifen.
