# Supplementary material for: Administration of Secretome Derived from Human Mesenchymal Stem Cells Induces Hepatoprotective Effects in Models of Idiosyncratic Drug-Induced Liver Injury Caused by Amiodarone or Tamoxifen
Source: Cells. 2023 Feb 16;12(4):636. doi: 10.3390/cells12040636 (PMC9954258; doi:10.3390/cells12040636)
Supplement: Supplementary file 1 [file cells-12-00636-s001.zip › Figure S8.pdf]

(a)

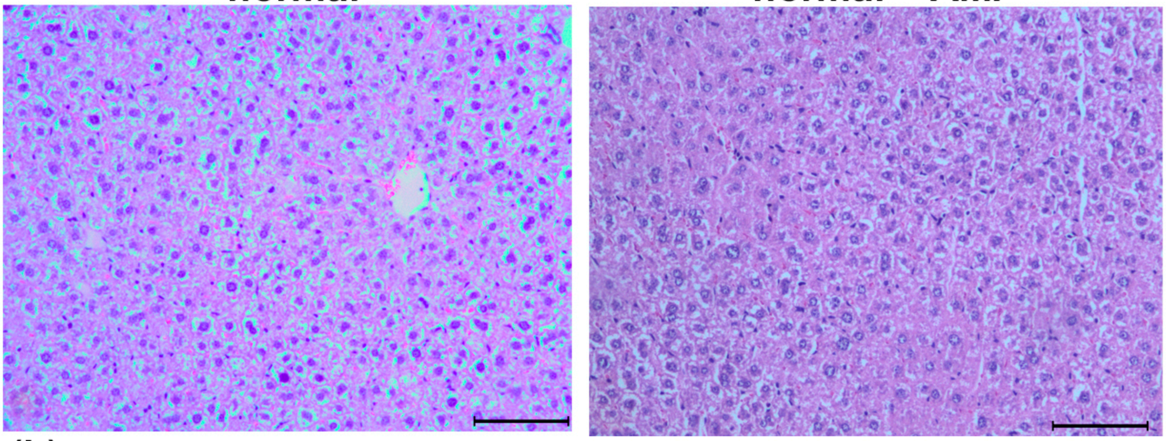

(b)

	Body weigth (g)	Blood glucose (mg/dl)	Serum triglyeryde (mg/dl)	Serum cholesterol (mg/dl)	Alanine aminotransferase (U/L)	Aspartete aminotransferase (U/L)
normal	26.8 ± 0.8	120 ± 25	52 ± 22	63 ± 10	32.0 ± 10.5	120 ± 30
normal + AMI	25.7 ± 1.5	110 ± 35	55 ± 19	70 ± 15	30.3 ± 13.8	139 ± 45

Figure S8: Histological and biochemical characterization of normal mice treated with AMI.

Male mice fed on a regular diet were treated daily with saline solution (normal group) or AMI (40 mg/kg) (normal + AMI), for four weeks. **(a)** Representative micrographs of liver sections stained with hematoxylin and eosin are shown. Scale bars represent 100 μm. **(b)** Biochemical examination of plasma from each experimental group (n=8).
