# Supplementary material for: Administration of Secretome Derived from Human Mesenchymal Stem Cells Induces Hepatoprotective Effects in Models of Idiosyncratic Drug-Induced Liver Injury Caused by Amiodarone or Tamoxifen
Source: Cells. 2023 Feb 16;12(4):636. doi: 10.3390/cells12040636 (PMC9954258; doi:10.3390/cells12040636)
Supplement: Supplementary file 1 [file cells-12-00636-s001.zip › Figure S9.pdf]

(a)

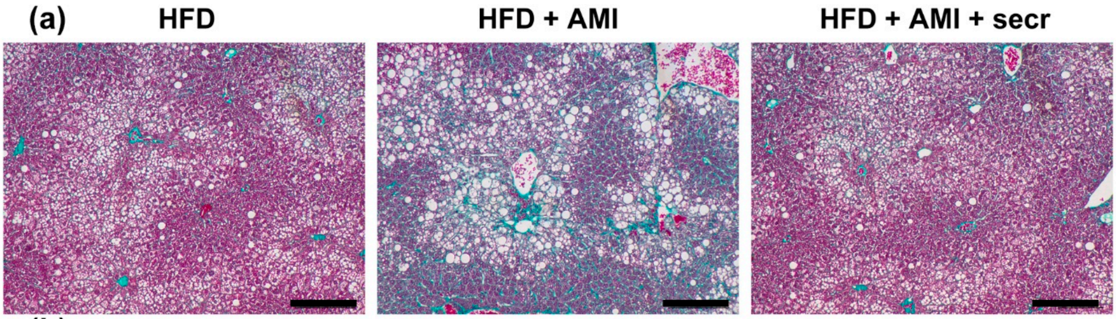

(b)

	Body weigth (g)	Blood glucose (mg/dl)	Serum triglerydе (mg/dl)	Serum cholesterol (mg/dl)	Alanine aminotransferase (U/L)	Aspartete aminotransferase (U/L)	Serum albumin (g/dl)
HFD	53.6 ± 4.1	152.2 ± 31	78 ± 12	140 ± 20	115 ± 31	125 ± 23	2 ± 0,2
HFD+AMI	51.8 ± 5.2	141.6 ± 33	68 ± 15	128 ± 25	84.9 ± 34	110 ± 34	2 ± 0,15
HFD+AMI+sec	49.9 ± 5.3	151.9 ± 20	75 ± 22	145 ± 18	68.1 ± 27.9	83 ± 26	1,9 ± 0,18

Figure S9: evaluation of liver fibrosis and biochemical profile in obese mice treated with AMI alone or AMI plus MSC secretome.

Male mice were fed a high-fat diet (HFD) for 30 weeks and were divided into three groups. The first group was treated with a weekly *i.p.* administration of saline solution (HFD group) for four weeks while the other two groups were treated daily with AMI (40 mg/kg), *i.p.*, for four weeks. At the end of each week, the second and third groups received by the tail vein 200 µl of saline solution (HFD+AMI group) or 200 µl of MSC secretome (HFD+AMI+secr) respectively. All mice were sacrificed 72 hours after the last treatment administration. **(a)** Representative photographs of liver sections with Masson´s trichrome to evaluate collagen fibers deposition. Scale bars represents 200 µm. **(b)** Results of biochemical analyses performed on plasma are shown for each experimental group (n=8).
