# Supplementary material for: Administration of Secretome Derived from Human Mesenchymal Stem Cells Induces Hepatoprotective Effects in Models of Idiosyncratic Drug-Induced Liver Injury Caused by Amiodarone or Tamoxifen
Source: Cells. 2023 Feb 16;12(4):636. doi: 10.3390/cells12040636 (PMC9954258; doi:10.3390/cells12040636)
Supplement: Supplementary file 1 [file cells-12-00636-s001.zip › Table S1.pdf]

**Table S1. RT-qPCR specific primers and characteristics of amplicons.**

| Gene | Nº GenBank access | Sense (5'→3') | Antisense (5'→3') | Amplicon | |
|---|---|---|---|---|---|
| | | | | size (bp) | Tm (ºC) |
| *human TNF-α* | NM_000594.4 | GAGGCCAAGCCCTGGTATG | CGGGCCGATTGATCTCAGC | 91 | 81 |
| *human IL-6* | NM_001371096 | CCAGAGCTGTGCAGATGAGT | AGCTGCGCAGAATGAGATGA | 174 | 80 |
| *human iNOS* | NM_000625.4 | TTCAGTATCACAACCTCAGCAAG | TGGACCTGCAAGTTAAAATCCC | 207 | 81 |
| *human CYP1A1* | NM_000499.5 | TCGGCCACGGAGTTTCTTC | GGTCAGCATGTGCCCAATCA | 141 | 82 |
| *human CYP1A2* | NM_000761.5 | CTGGGCACTTCGACCCTTAC | TCTCATCGCTACTCTCAGGGA | 99 | 79 |
| *human CYP2B6* | NM_000767.5 | TTCACGGTACACCTGGGAC | ATCACACCATATCCCCGGAAG | 146 | 83 |
| *human CYP2C9* | NM_000771.4 | CCTCTGGGGCATTATCCATC | ATATTTGCACAGTGAAACAT | 137 | 75 |
| *human CYP2C19* | NM_000769.4 | GGAAAACGGATTTGTGTGGGA | GGTCCTTTGGGTCAATCAGA | 109 | 76 |
| *human CYP2D6* | NM_001025161.3 | ACCAGGCTCACATGCCCTA | TTCGATGTCACGGGATGTCAT | 104 | 81 |
| *human CYP2E1* | NM_000773.4 | ATGTCTGCCCTCGGAGTCA | CGATGATGGGAAGCGGGAAA | 127 | 85 |
| *human CYP3A4* | NM_001202855.3 | AAGTCGCCTCGAAGATACACA | AAGGAGAGAACACTGCTCGTG | 174 | 77 |
| *human CYP7A1* | NM_000780.4 | GCAATTTGGTGCCAATCCTCT | GCACAACACCTTATGGTATG | 133 | 74 |
| *human Ribosomal protein L13a* | NM_001270491.2 | CGTGCGTCTGAAGCCTACAA | TGTCACTGCCTGGTACTTCC | 82 | 79 |
| *human GADPH* | NM_001357943.2 | CTGGGCTACACTGAGCACC | AAGTGGTCGTTGAGGGCAATG | 101 | 81 |
| *mouse GADPH* | XM_001474390 | ACTCCACTCACGGCAAATTC | TCTCCATGGTGGTGAAGACA | 171 | 88 |
| *mouse α-smooth muscle actin* | NM_007392 | TCAATGTCCCCGCCATGTATGT | AAGGAATAGCCACGCTCAGTCA | 220 | 89 |
